# Brain-derived neurotrophic factor amplifies neuron-intrinsic programs to enhance axonal regeneration in human motor neurons

**DOI:** 10.1126/scisignal.adx6752

**Published:** 2026-04-14

**Authors:** Jose Norberto S. Vargas, Anna-Leigh Brown, Kai Sun, Cathleen Hagemann, Bethany Geary, David Villarroel-Campos, Sam Bryce-Smith, Matteo Zanovello, Madeline Lombardo, Stan Majewski, Andrew Tosolini, Maria Secrier, Matthew J. Keuss, Andrea Serio, James N. Sleigh, Pietro Fratta, Giampietro Schiavo

**Affiliations:** 1 Department of Neuromuscular Diseases and UCL Queen Square Motor Neuron Disease Centre, UCL Queen Square Institute of Neurology, University College London, London, UK; 2 UK Dementia Research Institute, University College London, London, UK; 3 Department of Basic and Clinical Neuroscience, King’s College London, London, UK; 4 UK Dementia Research Institute, King’s College London, London, UK; 5 The Francis Crick Institute, London NW1 1AT, UK; 6 MRC Protein Phosphorylation and Ubiquitylation Unit, School of Life Sciences, University of Dundee, Dow Street, Dundee DD1 5EH, Scotland, UK; 7 Department of Genetics, Evolution and Environment, UCL Genetics Institute, University College London, London, UK

## Abstract

The cell-intrinsic capacity of neurons to regenerate axons requires widespread coordination of the transcriptome, activation of multiple kinases, and reorganization of the cytoskeleton. Axonal repair is also influenced by extrinsic activating factors, such as neurotrophins. Here, we report that brain-derived neurotrophic factor (BDNF) amplifies multiple neuron-intrinsic programs to foster axonal regeneration in human motor neurons. Through metabolic RNA sequencing and phosphoproteomic profiling, we elucidate BDNF signalling and its role in axonal regeneration. We discover that BDNF controls RNA stability and transcriptional programs that converge with regeneration-associated gene (RAG) sets. We further unveil that BDNF governs the phosphorylation of multiple proteins essential for cytoskeletal dynamics, a major determinant of effective nerve regeneration. Using compartmentalized neuronal cultures, we demonstrate that the regeneration driven by BDNF depends on the axon-specific activation of ERK/RSK/S6K kinase pathway. This highlights the crucial impact of subcompartment-specific kinase activation by neurotrophins in driving axonal regeneration. We propose a model in which BDNF augments neuron-intrinsic pathways to drive axonal regeneration in human motor neurons.

## Introduction

Nerve regeneration involves neuron-intrinsic pathways, as well as extrinsic activating signals, such as those provided by neurotrophins (1–3). The neurotrophin brain-derived neurotrophic factor (BDNF) is essential for motor neuron axon development and regeneration, as well as neuromuscular junction (NMJ) maintenance (3). At the NMJ, BDNF is released by the skeletal muscle and Schwann cells. BDNF then binds its cognate receptor, tropomyosin receptor kinase B (TrkB), on motor neurons, resulting in local and long-range pro-survival signalling important for nerve repair (3, 4). Conditional deletion of TrkB in neurons severely impedes peripheral nerve regeneration (5) and lentiviral transduction of TrkB enhances the regeneration of corticospinal axons (6), demonstrating the crucial role of BDNF in axon regeneration. Furthermore, dysfunctional neurotrophic support is an underpinning pathomechanism for motor neuron diseases and peripheral neuropathies (7), further underscoring its importance for axonal maintenance.

BDNF is known to promote pro-survival gene expression through the activation of several transcription factors. Effective gene expression requires the synchronisation of RNA transcription, stability, and decay processes. Whether neurotrophins regulate not just transcription, but also control other aspects of RNA dynamics, such as stability and degradation, is currently unknown. Indeed, the regulation of the axonal and dendritic transcriptome, which is crucial for their function, can be shaped by the stability of RNAs (8). Advances in RNA sequencing methodologies, such as metabolic labelling approaches, allow for the direct observation of rates of RNA transcription and/or degradation (9). Still, to the best of our knowledge, these approaches have not been utilised to assess RNA metabolism governed by neurotrophins. Importantly, the transcriptional outputs of BDNF in human motor neurons relevant for axonal regeneration are not fully elucidated.

Another crucial gap in our understanding of BDNF/TrkB signal transduction pathway is the lack of deep profiling of the phosphorylation landscape induced by BDNF activation, particularly in human motor neurons. It is well-established that several kinases are activated by BDNF, such as PI3K/AKT and MAPK pathways (10), yet a comprehensive characterization of BDNF-activated kinases, and their role in axonal regeneration in human neurons remains uncharted. For instance, the AGC-family kinase RSKs, which are activated by various growth signals through MAPK, have recently been shown to act as positive regulators of axonal regeneration (11, 12). Another AGC kinase, S6K, is also involved in axonal regeneration (13). However, the interplay of BDNF with RSK and S6K and the downstream substrates that govern axonal regeneration is currently unclear. The activation of specific kinases by BDNF plays a key role in modulating the morphogenesis of several neuronal sub-compartments, such as growth cones, synapses, dendrites and axons, all of which depend on cytoskeletal remodelling (14, 15). Indeed, axonal regeneration requires the dynamic restructuring of the cytoskeletal architecture (16). The organisation and function of the latter is, in part, dictated by microtubule-associated proteins (MAPs) and actin-binding proteins (17). Importantly, the phosphorylation of MAPs and actin-binding proteins regulates their binding to microtubules and motor proteins, thereby modulating cytoskeletal-related processes in a spatiotemporal manner (18). Thus, although we know that multiple kinases are activated by BDNF, we do not yet fully understand the specific phosphorylation substrates that enable BDNF to reorganise the cytoskeleton *en masse* to control neuronal architecture and axonal regeneration.

Acute versus gradual increases in the concentration of BDNF lead to different morphological changes, as well as kinase activation patterns in hippocampal neurons, highlighting kinetics as an important determinant of BDNF signalling (15). In addition to signalling kinetics, the activation of BDNF in various neuronal compartments also triggers differential signalling and phenotypic outputs. However, it is currently unknown whether the specific BDNF-dependent activation of kinases specifically either in the soma or axon can have distinct effects on axonal outgrowth and regeneration. Both the concentration and the spatial activation of BDNF receptors may be crucial for the various roles of this neurotrophin in the morphological upkeep of neurons, which is linked to which kinases are activated within distinct neuronal subcompartments. Given that neurotrophic-based therapies are currently being developed for ameliorating axonopathies, the subcellular effects of BDNF are highly relevant for determining the appropriate mode of delivery.

In this study, we unravel the RNA and proteomic programs controlled by BDNF through a combination of metabolic sequencing and phosphoproteomic analysis. We find a striking convergence of neuron-intrinsic regeneration pathways with those that are controlled by BDNF. We show that BDNF promotes global hypertranscription, which is a hallmark of regenerating tissues. Further, we show that BDNF governs the posttranscriptional tuning of steady-state RNA levels by altering RNA stability. Importantly, we uncover that the genetic regulatory networks controlled by BDNF overlap with those involved in promoting axonal outgrowth and repair. We further reveal that BDNF kinase signalling reshapes the phosphorylation pattern of cytoskeletal proteins, particularly of structural MAPs and actin-binding proteins. Through phosphoproteomic profiling of BDNF signalling and axonal regeneration assays using compartmented axon-specific kinase inhibition, we discover that the specific activation of ERK-RSK-S6K pathway in axons is required for BDNF to enhance axonal regeneration in human motor neurons.

Thus, we report a comprehensive characterisation of the transcriptomic and phosphoproteomic programs controlled by BDNF signalling. These findings provide novel molecular insights as to how an extrinsic growth-promoting signal, such as BDNF, drives neuron-intrinsic regeneration programs in human motor neurons.

## Results

### Assessing RNA dynamics with SLAM-seq in human motor neurons

To study the role of BDNF in human motor neurons, we used the genetically-engineered human iPSC-derived lower motor neurons (i^3^ LMNs) expressing *NGN2, ISL1*, and *LHX3* under a doxycycline-inducible promoter (fig. S1A-D; Fernandopulle et al., 2018). Post-differentiation, i^3^ LMNs expressed ChAT and Hb9, which are two canonical motor neuron markers (fig. S1C-D, F), and responded to BDNF through the activation of well-known downstream kinases MAPK-ERK1/2 (referred as ERK from here), AKT, and S6K (fig. S1E).

**Figure 1:**
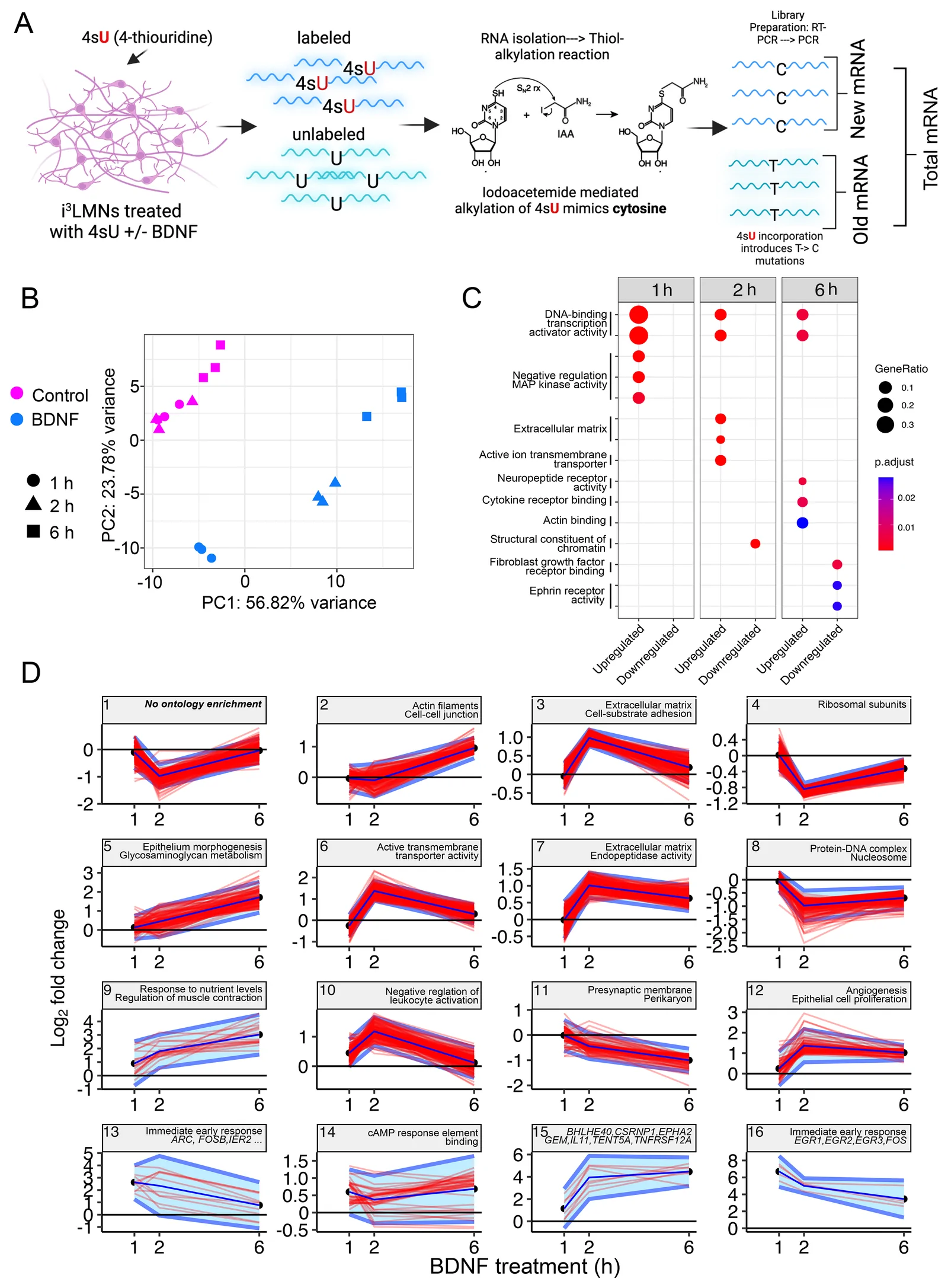
BDNF-driven transcription in human motor neurons regulates time-resolved functional networks A) Schematic of SLAM-sequencing pipeline. Neurons were treated with 100 µM 4sU uridine analogue which is integrated into newly transcribed RNA. After RNA extraction, RNA was incubated with iodoacetamide, which promotes the thiol-alkylation of 4sU. Before standard RNA-sequencing, RNA is reverse-transcribed to cDNA; during this process the thiol-alkylated 4sU will base pair with guanine rather than adenine, thus mimicking cytosine. When the RNA sequencing is mapped back to the genome, there will be characteristic T > C mismatches in the reads produced during the labelling period which can be used to infer the ratio of new to total RNA. N=3 biological replicates per condition and labelling time. B) Total RNA principal component analysis showing separation of control (pink) and BDNF treated (blue) lower motor neurons by both treatment condition and time. Close clustering of control samples along PC2 suggests that 4sU treatment had minimal effect on total gene abundance C) Gene-ontology analyses for genes with increase and decrease in abundance across 1, 2, 6 h BDNF treatment collapsed for semantic similarity. Differentially expressed genes on total RNA called with an adjusted p-value < 0.1 and the abs(log_2_ fold change) > 0.75 estimated by DESeq2(65). D) Differential gene expression hierarchically clustered by log_2_ fold change across BDNF treatment showing mean and individual gene cluster expression with cluster GO terms/gene names. Individual gene expression as red lines, mean expression of cluster dark blue lines, and twice width cluster standard deviation as light blue boundaries. Genes which were never called as differentially expressed at any of the three timepoints and with a baseMean < 15 were removed prior to clustering.

To dissect the transcription kinetics driven by BDNF in i^3^ LMNs, we performed SLAM-seq (20) at 1, 2, and 6 hours of post-treatment with BDNF (Fig. 1A). SLAM-seq provides information on total RNA abundance to assess differentially expressed genes (DEGs), in addition to providing a direct readout of RNA transcription in the same sequencing experiment. From these measurements, RNA degradation/destabilisation can be inferred by comparing ratios of newly transcribed RNA and total RNA levels, thus allowing us to investigate the effects of BDNF signalling on RNA metabolism.

### BDNF controls functionally distinct and time-resolved transcriptional networks

First, we assessed DEGs upon BDNF treatment in human motor neurons. Principal component analysis (PCA) on total RNA revealed a clear separation between BDNF-treated and control samples (Fig. 1B). Immediate early response genes, reflecting a rapid but transient response to BDNF, are the main drivers of variance in condition- and time-correlated principal components (fig. S2A). We detected the high expression of *EGR1, FOS, JUNB*, and *ARC*, which are known early-responsive genes to BDNF signalling, confirming the validity of both the treatment and sequencing (fig. S2B). Overall, we found that the number of BDNF-dependent DEGs increased from 1 (58 genes) to 2 hours (2,131 genes), followed by a reduction at 6 hours (604 genes) (fig. S2B-D; Data File S1), suggesting that gene expression driven by extrinsic BDNF exhibits autoregulation at longer timepoints.

To functionally map the transcriptional output of BDNF, we next performed gene-ontology (GO) enrichment for DEGs across labelling times (Fig. 1C). DNA-binding transcription activators are significantly enriched across all timepoints, consistent with the role of BDNF in transcriptional control. Negative regulators of the MAPK pathway *DUSP1, DUSP5*, and *DUSP6* had transiently increased abundance at 1 hour (Fig. 1C; fig. S2B-D), potentially indicating a negative feedback transcriptional response to attenuate sustained ERK signalling. DEGs related to extracellular matrix (ECM) and neuronal cytoskeletal components, including actin-binding factors, are enriched at later time points (Fig. 1C; Data File S2). At 2 hours, chromatin structural genes had decreased abundance, perhaps reflecting BDNF’s effect on chromatin accessibility to induce gene expression (21). Genes encoding members of the EPHR receptor tyrosine kinase family (*EPHA4*/*EPHA3*), which are mainly axon-repulsive, had decreased abundance by 6 hours, suggesting that prolonged BDNF treatment can negatively regulate axonal repulsion (Fig. 1C; Data File S2). Intriguingly, reduction of *EPHA4* increases peripheral nervous system regeneration (22) and lower *EPHA4* expression has been shown to protect against axonal degeneration in both animal models and ALS patients (23).

BDNF/TrkB signalling is tightly regulated, with its effects on neuronal morphology and differentiation varying based on treatment duration and BDNF concentration (15). Furthermore, BDNF-driven gene expression in mouse and human cortical neurons has been shown to exhibit distinct early and late transcriptional responses (21). To explore this phenomenon in motor neurons, we analysed temporal and functional RNA expression downstream of BDNF signalling at different time points using hierarchical clustering and identified 16 distinct expression clusters (Fig. 1D; Data File S2). Immediate early response genes (clusters 13 and 16) showed a rapid increase in total RNA abundance, returning to baseline by 6 hours, indicating the validity of this approach. Genes related to neuronal morphology displayed delayed increases in total RNA, ECM-related genes (clusters 3 and 7) peaked at 2 hours, while actin filaments (cluster 2) remained stable until an increase in total RNA at 6 hours. Clusters involving ribosomal subunits (cluster 4) and DNA-binding components (cluster 8) had decreased total RNA abundance at 2 hours but began to rebound by 6 hours. A small cluster (cluster 15) exhibited a sustained increase in total RNA abundance (Fig. 1D; Data File S2). These data show highly specific clusters of genes that respond in a coordinated manner to BDNF. This indicates that the temporal activation of TrkB regulates specific cellular processes through functionally defined transcriptional responses. Of interest, we observed gene clusters comprising ECM, cell adhesion, and translation components, all of which are implicated in axonal maintenance. Taken together, these findings show that BDNF drives time-dependent and functionally distinct expression patterns in human motor neurons.

### BDNF increases global rates of transcription in human motor neurons

**Figure 2:**
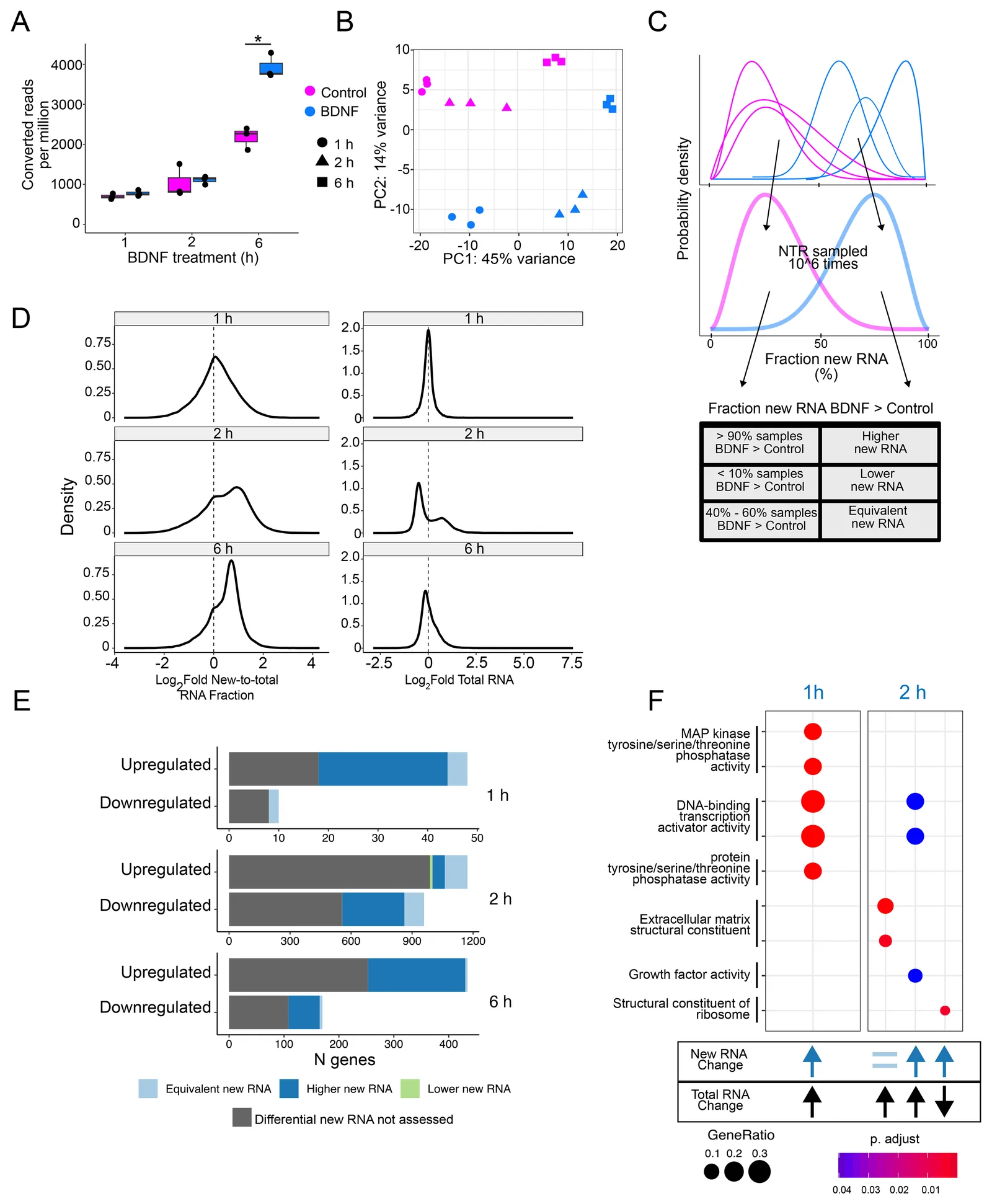
BDNF coordinates global transcription and widespread changes in RNA stability A) SLAM-seq converted reads with at least 2 characteristic T>C mismatches per million of mapped reads at 1, 2, and 6 h of 4sU treatment in control and BDNF treated i3 LMN. Schematic of pipeline in Fig. 1A. N=3 biological replicates per condition and labelling time. B) Converted reads principal component analysis. C) Schematic of Monte-Carlo sampling approach used to determine condition specific new-to-total RNA ratio (NTR). Beta-distributions on the NTR for a gene for each of N = 3 biological replicates at each time point were estimated by GRAND-SLAM. Condition-specific beta distributions were created by merging the sample beta-distributions with each sample weighted inversely by its variance. Condition NTR was calculated by performing one million samples from each condition’s beta distribution, and differential labelling was determined by comparing the difference between each of the 1 million samples. D) Transcriptome-wide distribution of the log2 fold change of condition specific NTR RNA fraction (left) and the log2 fold change of total RNA. Of genes which met strict NTR cutoff (90% credible interval < 0.2), by 2 h the majority of genes had a higher NTR ratio with BDNF treatment (1 h - 1,392 of 6,169 genes; 2 h - 6,858 of 8,835 genes; 6 h - 8,246 of 8,909 genes). E) Categorization of genes by BDNF effect on total RNA abundance and NTR across treatment times. Stabilised - increase abundance total RNA, equivalent or lower new RNA; 1 h - 4 genes; 2 h - 123 genes; 6 h - 4 genes. Destabilisation - decrease abundance total RNA, higher new RNA; 1 h - 0 genes; 2 h - 306 genes; 6 h - 57 genes, and transcriptionally increased - increase abundance total RNA, higher new RNA; 1 h - 26 genes; 2 h - 61 genes; 6 h - 177. F) Gene-ontology enrichment for genes categorised in (E). No significant GO terms were found at 6 h treatment time. Significant GO terms using an adjusted p-value < 0.05.

Next, we explored the transcription kinetics driven by BDNF in human motor neurons. SLAM-seq relies on the incorporation of 4-thiouridine (4sU) into nascently transcribed RNA. RNA is extracted and treated with iodoacetamide, leading to thiol-alkylation of 4sU. When RNA is mapped to the genome, this results in T>C conversions in the nascently transcribed RNA(20, 24). We first measured the effect of BDNF on the direct readout of transcription during SLAM-seq (reads with at least two characteristic T>C mismatches, termed converted reads). Converted reads rose with 4sU labelling time in both control and BDNF-treated motor neurons. By 6 hours, BDNF-treated samples had significantly more converted reads, indicative of increased transcriptional rates (Fig. 2A). PCA on converted reads revealed distinct clustering of treatment groups based on time and BDNF treatment (Fig. 2B). To assess transcriptional rate changes driven by BDNF, we used a Monte-Carlo simulation to calculate a new-to-total RNA ratio (NTR) for each gene that met a strict NTR cutoff (90% credible interval < 0.2)(25). Briefly, for each gene we modelled the condition-specific NTR by combining each sample probability distribution to build a beta distribution varying from 0 to 1. Then, we sampled 1 million times from both the control and BDNF probability distributions to assess whether or not the NTR was significantly different between the two conditions (Fig. 2C). We found that BDNF increased global transcription rate, particularly at 6 hours, which did not correspond to a rise in steady-state RNA abundance (Fig. 2D). This increase in global transcription triggered by BDNF is akin to “hypertranscription,” which is the widespread amplification of transcription in specific cellular contexts, such as during neuronal maturation, cellular growth, and regeneration (26). Crucially, these processes are regulated by BDNF in the central and peripheral nervous system (27).

### BDNF signalling alters RNA stability in human motor neurons

We next investigated how BDNF-dependent gene regulation is shaped through transcription and RNA stability mechanisms. We grouped genes by both their change in total RNA levels and newly synthesised RNA rates to reveal genes with patterns of stabilisation, destabilisation, and transcriptional upregulation (Fig. 2E; Data File S1). At 1 and 6 hours of BDNF treatment, most genes with increased total RNA abundance showed signs of transcriptional increase with higher new RNA fractions (Fig. 2E; Data File S1). We also identified a group of RNAs with decreased abundance, but which were highly transcribed 2 hours post-BDNF treatment (Fig. 2E), indicating a large-scale degradation of RNAs by BDNF after 2 hours of treatment, despite the hypertranscription observed (Fig. 2D). Prolonged BDNF treatment in iPSC-derived cortical neurons and mouse GABAergic neurons also lead to widespread downregulation of RNAs (21, 28). These findings suggest sustained treatment of BDNF can drive RNA degradation, although the mechanism and function of this phenomenon are currently unclear.

We then categorised genes by the effect of BDNF on the ratio of total to new RNA across treatment timepoints and performed GO analyses (Fig. 2F; Data File S2). At 1 hour of treatment, RNA regulation was primarily driven by transcriptional upregulation, affecting MAPK pathway inhibitors, such as *DUSP1, DUSP5*, and *DUSP6*, along with early response genes like *ARC, FOS*, and the regeneration-associated transcription factor *ATF3* (Fig. 2F; Data File S2). We validated the transcriptional upregulation of *FOS*, a known BDNF-activated gene, which showed a 1.28 log_2_fold increase in NTR and a 7.56 log_2_fold increase in total RNA abundance in our SLAM-sequencing experiment at 1 hour (Data File S2) by RT-qPCR. We confirmed a significant increase in *FOS* RNA abundance which was completely abrogated by the transcription inhibitor actinomycin D (fig. S2E). At 2 hours, transcriptional upregulation also increased the expression of genes associated with growth factor activity, such as the regulators of neurite growth *VEGFA, IL11*, and *JAG1* (Fig. 2F; Data File S2). We also observed a group of genes that showed increased total RNA abundance, without increases in NTR, suggesting possible RNA stabilisation mechanisms. Notable examples of these genes are those encoding ECM structural proteins (Fig. 2F; Data File S2).

Amidst the robust increase in the newly transcribed RNA ratio for several genes driven by BDNF, some of these genes exhibited a paradoxical decrease in total RNA levels (Fig. 2F; Fig. 1D; Data File S2). BDNF activates transcription and simultaneously initiates the translation of hundreds of mRNAs (29). Recent work indicates that increases in ribosomal flux, the rate at which ribosomes initiate translation on an mRNA, can destabilise actively translating mRNAs (30). Thus, it is possible that some mRNAs downregulated by BDNF may also be concomitantly translated. To test this, we analysed the abundance of mRNA encoding the dynein subunit *DYNC1I2*, which had significantly decreased total RNA abundance with BDNF at 2 hours. We treated i^3^ LMNs with BDNF and the translational inhibitor cycloheximide for 2 hours and performed RT-qPCR. The RNA abundance of *DYNC1I2* significantly decreased upon BDNF treatment, in line with our sequencing data (fig. S2F). Importantly, co-treatment of BDNF with cycloheximide inhibited the decrease in abundance, indicating that the destabilisation of at least some mRNAs by BDNF is potentially due to simultaneous translation. In line with our findings, BDNF has been found to increase ribosomal protein abundance through mTOR-dependent translation in neurons (31, 32). This increased translation of ribosome components, along with the evidence that elevated translational rates destabilise mRNAs (30), may partly explain the concomitant hypertranscription and instability of ribosomal genes in our data sets (Data File S2). Nevertheless, a direct comparison of ribo-sequencing with metabolic RNA datasets is required to ascertain the full extent of translation-mediated mRNA destabilisation induced by BDNF. Thus, we show in human motor neurons that BDNF triggers hypertranscription, while also modulating the expression levels of many genes post-transcriptionally, potentially through changes in RNA stability.

### BDNF drives the expression of regeneration-associated genes and injury-inducible transcription factors

**Figure 3:**
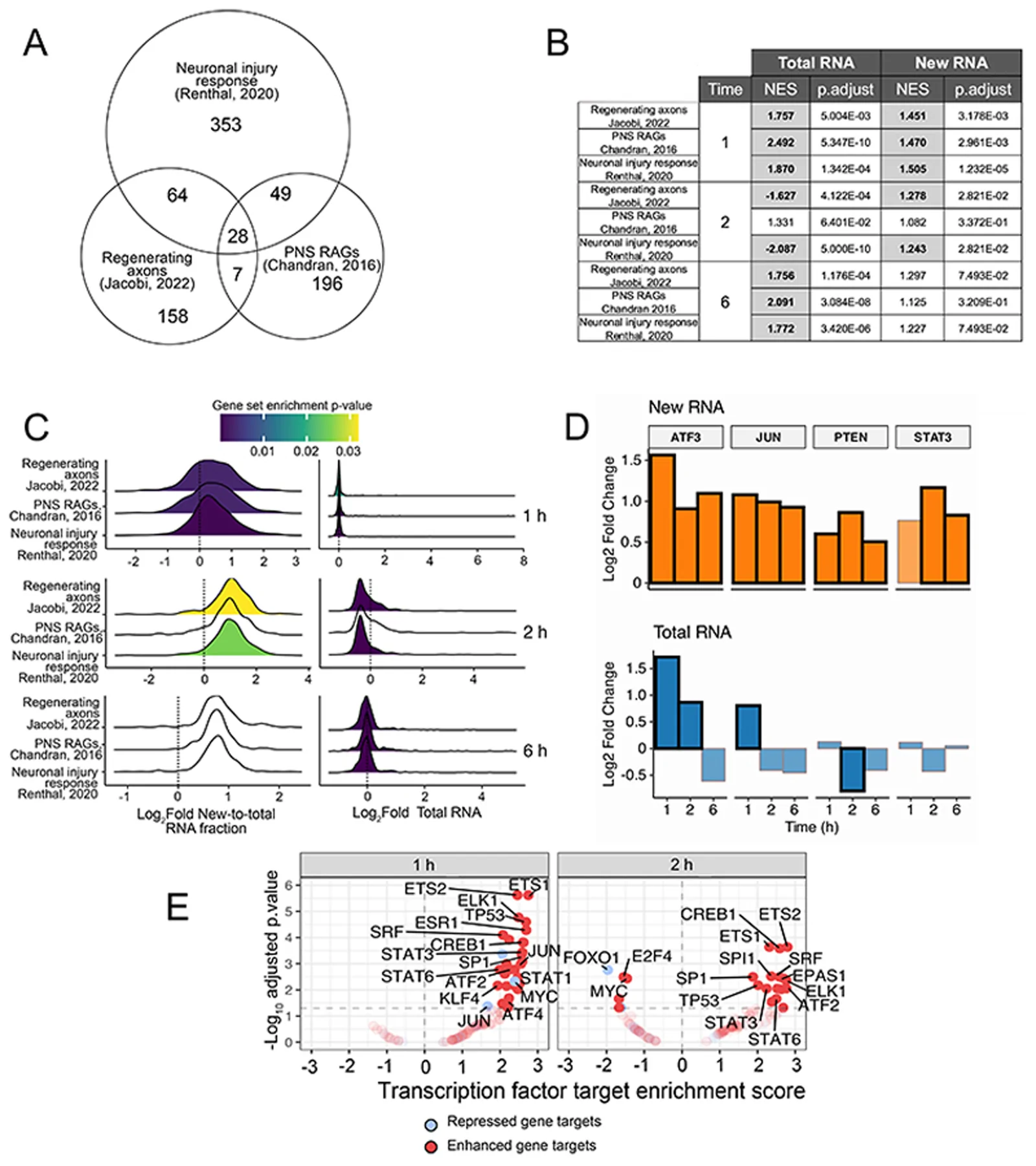
BDNF drives regeneration-associated transcriptional programs in human motor neurons A) Number of genes and overlap between three different gene sets defining neuronal injury response. Gene sets are originally derived from *Rattus norvegicus* (PNS RAGs, Chandran 2016) and Mus musculus (Regenerating axons, Jacobi 2022 and Neuronal injury response, Renthal 2020) and were first mapped to their human orthologs using gprofiler2 (69). B) Normalised enrichment score (NES) and adjusted p-value for the log_2_ fold change of total RNA and NTR on neuronal injury response gene sets in C. Bolded and highlighted enrichments are significant at adjusted p-value < 0.05. C) Distribution of the log_2_ fold NTR and total RNA for axonal regeneration sets. Area under curve coloured: significant; blank: non-significant. Colour refers to the adjusted p-value of the gene-set enrichment analysis of that gene set. NES as listed in B. Significant gene set enrichment analysis (GSEA) with an adjusted p-value < 0.05. D) Total (orange) and new RNA (blue) log_2_ fold change across time for select known regeneration associated genes showing differing effects of BDNF on both abundance and RNA turnover across treatment time. Significant total RNA called with DESeq2 (65) and significant new RNA called using Monte-Carlo simulation described in Fig. 2A. E) GSEA transcription factor (TF) regulons from DoRothEA. Red - TF activates target genes; blue - TF represses target genes. Positive TF enrichment scores - target genes increase abundance. 6 h - only ETS1 activated targets were significantly enriched (NES = 2.037, adjusted p-value = 0.02069). Significant GSEA with an adjusted p-value < 0.05.

Injury-inducible genes are critical for axonal regeneration (2, 33). Given the role of BDNF in peripheral axon regeneration, we examined its effects on transcriptional programs specifically linked to neuronal injury. We used three high quality compendiums of regeneration-responsive genes (34–36) (Fig. 3A). We then performed Gene Set Enrichment Analysis (GSEA) on BDNF-induced changes in abundance and newly transcribed RNA in motor neurons (Fig. 3A,B). Strikingly, we found that BDNF-regulated transcripts overlap with regeneration-associated genes (RAGs). At 1 hour, all three datasets showed increased transcriptional rates and RNA abundance (Fig. 3B,C), suggesting BDNF rapidly increased the transcription of genes critical for neuronal injury response. However, at 2 hours, two gene sets showed a paradoxical pattern similar to what we observed for ribosomal protein genes: total RNA levels decreased while new RNA increased, suggesting active mRNA degradation despite ongoing transcription (Fig. 2C). By 6 hours, total RNA levels were enriched across all datasets, but new RNA enrichment was absent. Altogether, BDNF signalling rapidly increased a cluster of genes crucial for neuronal injury response even in the absence of axonal damage. BDNF may therefore increase the responsiveness of motor neurons to axonal injury.

We next investigated the expression patterns of three exemplar RAGs (*ATF3, JUN*, and *STAT3)*. We found that these RAGs were highly transcribed by BDNF as shown by a significant increase in NTR across all timepoints (Fig. 3D). We found *ATF3* and *JUN* are autoregulated at total RNA levels, since both had increased total RNA abundance at 1-2 hours. In contrast, the total levels *of STAT3* did not change amidst the high transcriptional rates (Fig. 3D). *PTEN*, a negative regulator of axonal outgrowth, shows increased levels of new RNA, even though we observed a decrease in total RNA abundance at 2 hours, suggesting the destabilisation of *PTEN* (Fig. 3D). These data indicate that BDNF regulates the expression of certain RAGs through RNA stability.

The cell-intrinsic capacity of neurons to respond to axonal insults requires the activation of injury-inducible transcription factors (2). To explore which transcription factors are putatively activated by BDNF in motor neurons, we performed GSEA with the DoRothEA database(37), which curates benchmarked transcription factor-target gene interactions (Fig. 3E). BDNF signalling activated multiple transcription factors at 1 and 2 hours (Fig. 3E). We did not detect any enriched TFs at 6 hours except for ETS1. We found the majority of transcription factors we identified as BDNF-responsive in motor neurons play a role in neuron-intrinsic regeneration pathways following axonal injury (Table 1). This analysis shows that BDNF signalling potentially fosters the activation of multiple injury-inducible genes sets, which are co-regulated by transcription factors essential for axonal outgrowth and regeneration.

Overall, through SLAM-seq and computational analyses of BDNF-responsive genes, we found that BDNF signalling triggers global hypertranscription and controls RNA stability. Furthermore, BDNF rapidly and transiently increases transcription of RAGs and a subset of cell-intrinsic neural repair TFs.

### BDNF reshapes the phosphorylation landscape of cytoskeletal-interacting proteins in human motor neurons

In addition to modulating transcriptional rates and TF activation, BDNF also synchronously activates several kinases. Given that many TFs are mobilized by phosphorylation, BDNF-activated kinases are interlinked to RNA regulation. For instance, in neurons BDNF activates CREB and STATs directly through ERK, both TFs being crucial for axonal regeneration (2). Indeed, axonal-specific activation of kinases after axonal injury is connected to TF activation in the soma in facilitating regeneration (38). Therefore, a compendium of the transcriptomic and phosphoproteomic programs by BDNF in motor neurons is required to understand the roles of BDNF in nerve regeneration.

**Figure 4:**
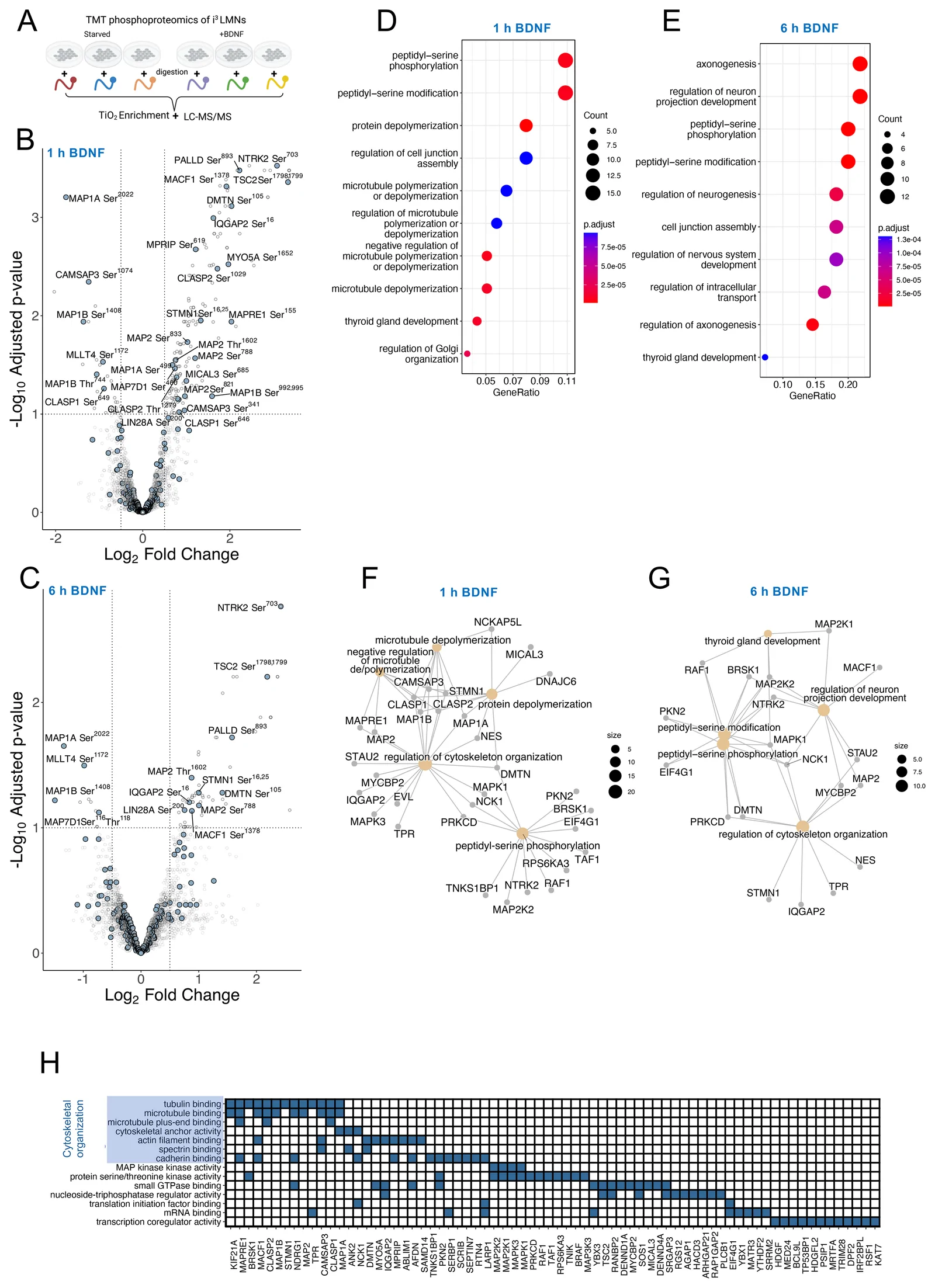
BDNF governs the phosphorylation landscape of cytoskeletal-binding proteins A) Schematic of tandem mass tagging (TMT) phosphoproteomics. Unique isobaric tags were added to control, 1 h, and 6 h BDNF-treated DIV7 i3 LMNs. N=3 biological replicates per condition (No BDNF, 1 h and 6 h 25 ng/mL BDNF). B-C) Volcano plot showing the fold change and adjusted p-values of the phosphorylation sites at 1 h (B) and 6 h (C) after BDNF (25 ng/mL) treatment. Each point represents a phosphosite. Blue dots represent microtubule associated proteins (MAPs) and known downstream targets of BDNF. Significantly differentially phosphorylated sites are labelled with protein name and position in the sequence. Dotted lines represent adjusted p-value 0.1 and log2 fold change +/-0.5. 148 significant phosphorylation hits at 1 h and 49 hits at 6 h of BDNF treatment (Data File S3). D-E) Dotplot showing gene-ontology (GO) enrichment on proteins with significant changes in phosphorylation at 1 h (D) and 6 h (E) after BDNF treatment. F-G) Gene-concept networks depicting the gene-GO relationships for proteins with increased phosphorylation at 1 h (F) and 6 h (G) after BDNF treatment. H) Gene-concept heatmap showing the overlap between significantly phosphorylated proteins after 1 h BDNF treatment. The shared gene-ontology terms, which are highlighted in the light blue box, are the GO terms related to cytoskeletal organisation.

All aspects of axonal repair rely upon precise changes to cytoskeletal dynamics that support the rebuilding of damaged axons. Cytoskeletal remodelling is determined by structural microtubule-associated proteins (MAPs) and actin-binding components (16, 39). BDNF has been shown to phosphorylate components of the cytoskeleton, however its substrates relevant to axonal regeneration have not been fully characterized. To address this, we performed tandem mass tag (TMT)-based quantitative phosphoproteomics in i^3^ LMNs upon BDNF incubation (Fig. 4A; Data File S3). To ensure signal transduction specificity, neuronal media was replaced with starvation media lacking other growth factor sources for 2 hours prior to BDNF treatment. After 1 and 6 hours of BDNF treatment, we confirmed that NTRK2/TRKB is the most phosphorylated protein identified, along with the well-established signalling nodes expected to be activated by BDNF, such as TSC2 and MAP2K1/MEK1 (Fig. 4B-C).

Crucially, we discovered striking changes in phosphorylation of several microtubule- and actin-binding proteins downstream of BDNF signalling (Fig. 4B-C). The robust enrichment of these proteins in our phosphoproteomic data (Fig. 4B-G) is crucial, since their phosphorylation status dictates their function (18). Delving deeper into the phospho-regulation of these proteins by BDNF, we found multiple phosphorylation and dephosphorylation events within a single MAP (Fig. 4B,C). For instance, both MAP1A and MAP1B showed phosphorylation and dephosphorylation at multiple sites (Fig. 4B,C). Several of these phospho-events were also time-dependent indicating complex phosphorylation patterns on structural MAPs in response to BDNF (Fig. 4B,C).

Other cytoskeletal-binding proteins that were significantly phosphorylated downstream of BDNF include: MAP2, MAP7D, MAPRE1/EB1, MACF1, CAMSAP3, CLASPs, PALLD, DNMT, MICAL3, MLLT4, NCKAP5L, and IQGAP2 (Fig. 4B-C,F-G). The functional diversity of these proteins include microtubule-stabilizing and -destabilizing, actin-microtubule crosslinking, microtubule plus and minus end-binding, all of which regulate the cytoskeletal organization of neurons and may be consequential for axonal regeneration (16–18).

Since we identified multiple cytoskeletal-binding proteins as phospho-substrates of BDNF, we sought to further characterise these events. To this end, we generated a sequence logo (40) of the significant phosphopeptides identified, and found that BDNF treatment primarily resulted in phosphorylation of serines followed by a proline at the +1 position (pS-P). Although not as pronounced as pS-P, the basophilic RXRXXpS/T (X = any amino acid) phospho-motif canonical to the AGC superfamily kinases (41) was also evident in our motif analysis (fig. S3A). Proline-directed kinase families include ERK, mTOR, and cyclin-dependent kinases, which are known downstream effectors to TrkB activation. When we focused our analysis on proteins with significant proline-directed serine phosphosites, we found an enrichment of cytoskeletal-binding proteins (fig. S3B). We validated by western blotting two proline-directed BDNF-dependent phosphorylation events on STMN1 Ser^25^, a microtubule destabilising enzyme, and Lin28a Ser^200^, an RNA-binding protein (RBP) known to enhance axonal regeneration (42) (fig. S3C-F). Both phosphorylation sites are driven by BDNF and inhibited by the addition of U0126, a well-established ERK inhibitor, indicating that BDNF triggered the phosphorylation of at least these two sites through ERK in human motor neurons (fig. S3C-F).

### BDNF phospho-substrates are enriched for regulators of cytoskeletal dynamics and axonogenesis

We next performed unbiased functional enrichment analyses on the significant BDNF-dependent phospho-events in i^3^ LMNs. At both 1 and 6 hours of BDNF treatment, we discovered an overrepresentation of the microtubule polymerization and depolymerization pathways (Fig. 4D-E). Additionally, pathways related to axonogenesis, neuron projection, and regulation of intracellular transport processes, all of which depend on cytoskeletal organization, were highly represented among BDNF substrates (Fig. 4D-E). Next, we created gene-concept networks of proteins with increased phosphorylation to better illustrate the enriched functional networks associated with BDNF phospho-signalling (Fig. 4F-G). The gene-concept networks revealed several signalling hubs with well-characterised functions on the neuronal cytoskeleton (Fig. 4F-G). These hits include BRSK1, which regulates neuronal polarity through Tau phosphorylation (43), and the kinase RPS6KA3/RSK2, which has been reported to enhance axonal regeneration (11). Additional hits were MYCBP2, an atypical E3 ligase important for regulation of Wallerian degeneration (44), and NCK1, which was found to stabilise neuronal actin dynamics (45). We also identified an understudied microtubule plus end-binding protein, NCKAP5L (46), as a novel phospho-substrate of BDNF (Fig. 4F-G). Thus, our unbiased computational approaches reveal that BDNF regulates not only the phosphorylation of many cytoskeletal components, but also of molecular pathways implicated in cytoskeletal dynamics.

Other pathways enriched by BDNF treatment were the regulation of MAPK/ERK signalling, translation initiation factor binding, mRNA binding, and transcription coregulation activity (Fig. 4H). The ALS-related RBP MATRIN3, known to regulate RNA stability, was also highly phosphorylated upon BDNF treatment (Data File S3). Other RBPs that regulate mRNA stability, such as STAU2, YBX1 and YTHDF2, were also identified as phospho-substrates of BDNF. Of note, YTHDF proteins are known positive regulators of axonal regeneration through regulation of RNA stability (47), and the ERK-dependent phosphorylation of YTHDF2 at serine 39, detected in our dataset (Data File S3), facilitates its RNA degradative function (48). These RBPs impact RNA metabolism in neurons and are potentially involved in regulating the post-transcriptional tuning of RNA levels induced by BDNF described herein; however, more work is required to validate this hypothesis (Fig. 2).

### BDNF phosphorylation events in motor neurons are enriched for ERK-RSK-S6K kinase motifs

**Figure 5:**
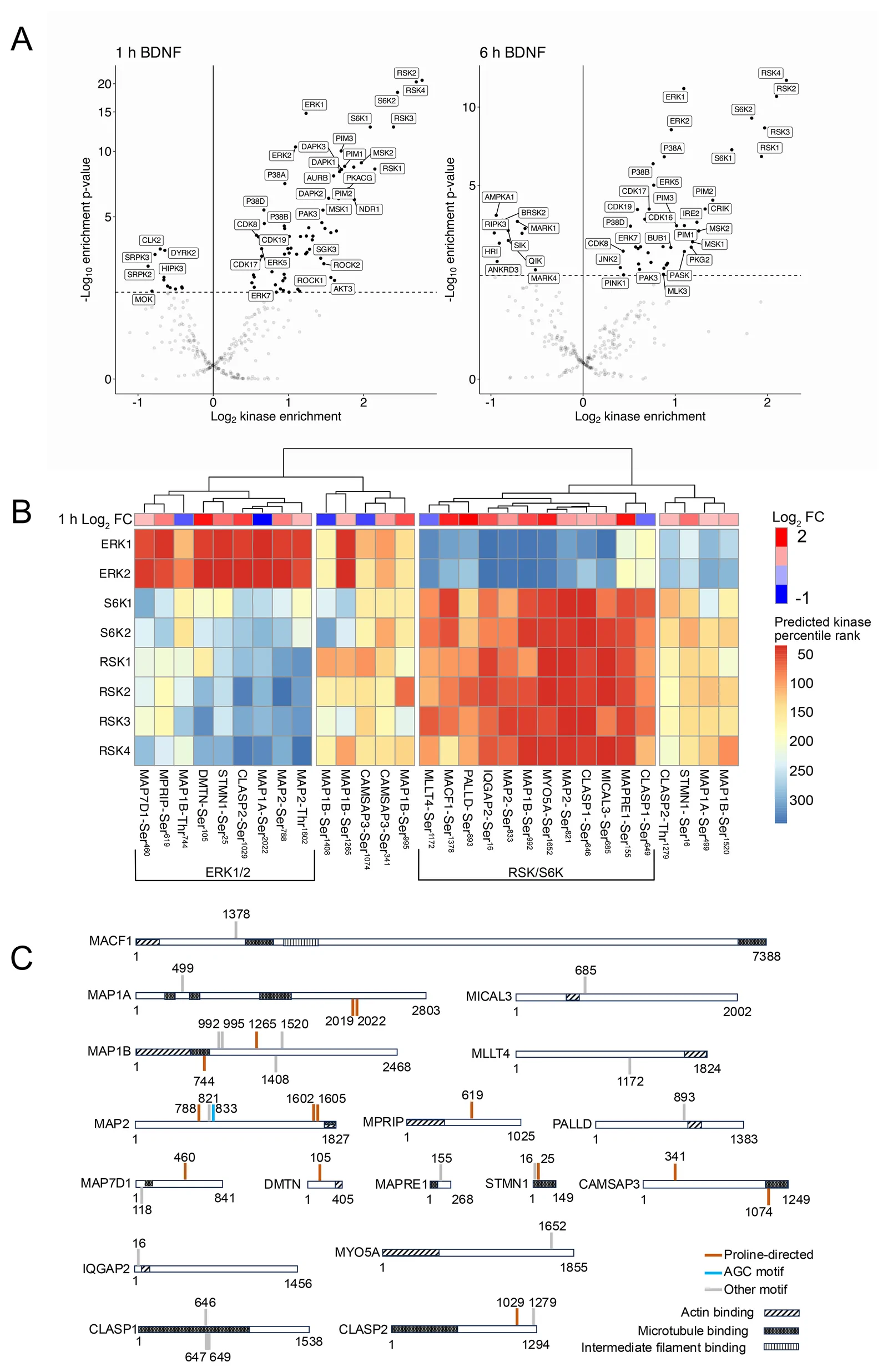
Kinase prediction of BDNF phosphosubstrates identifies ERK, S6K, and RSK targeting cytoskeletal proteins downstream of BDNF. A) Predicted kinase enrichment at 1 h and 6 h after BDNF treatment based on sequence of differentially phosphorylated sites. Dotted line shows significant enrichment at p-value < 0.01. B) ERK, S6K, and RSK kinase specificity on microtubule proteins at 1 h BDNF treatment. The heatmap represents the percentile rank of each kinase against the given peptide across the panel of 311 serine/threonine kinases (49). The upper ribbon shows the log_2_fold change of the phosphorylation after 1 h BDNF treatment. Sites have been hierarchically clustered based on kinase percentile ranks. C) Significant phosphorylation events on proline-directed (pS/TP) and AGC (RXRXXpS/T) motifs downstream of BDNF treatment on microtubule and actin-binding proteins showing proximity to cytoskeletal-binding regions.

Following up on our TMT phosphoproteomics experiment, we used a kinase prediction tool based on large-scale combinatorial synthetic peptide libraries, which can identify the substrate sequence specificity of 311 serine/threonine kinases (49). This kinase enrichment analysis predicts activated kinases by ranking the affinity of all kinases in the library towards the motifs of significant phosphorylation events with Fisher’s exact test. Using the phosphorylation events driven by BDNF as inputs, we found that AGC-family kinases, RSK/p90RSK and S6K/p70S6K, and ERK were predicted to be the top kinases phosphorylating substrates downstream to BDNF activation (Fig. 5A).

Next, we aimed to predict how likely any given kinase is to phosphorylate the significant motifs we identified. To this end, we analysed the percentile rank of each of the serine/threonine kinases, which indicates how likely a kinase is to phosphorylate any specific motif compared to other kinases (Data File S4). Given that cytoskeletal proteins are the most abundant phospho-substrates of BDNF, we performed hierarchical kinase clustering on the phosphorylated actin and microtubule interacting proteins based on the percentile rank score of ERK-RSK-S6K kinases (Fig. 5B). We identified four major clusters. A group of phospho-motifs, which includes the ERK-dependent STMN1 Ser^25^ site we validated biochemically (fig. S3C-D), showed high predicted specificity for ERK (Fig. 5B). We also found a cluster with high predicted specificity of RSK/S6K, potentially indicating direct phosphorylation of the sites by these kinases (Fig. 5B). The other clusters of cytoskeletal component sites showed low preference for ERK-RSK-66K (Fig. 5B). Crucially, many of the phospho-events on cytoskeletal proteins we detected are proximally or within the actin- and microtubule-binding regions of these proteins (Fig. 5C), hinting at a regulatory influence of BDNF on their cytoskeletal-binding properties. Thus, BDNF regulates the global phosphorylation of cytoskeletal-binding components, potentially through downstream activation of both ERK1/2 and RSKs and S6Ks kinase activity.

Taken together, these data indicate that the phosphorylation targets downstream of BDNF in human motor neurons are highly enriched for cytoskeletal components, as well as various signalling pathways involved in enhancing neuronal morphology and axonal regeneration. Kinase enrichment analyses reveal that these phosphorylation events are likely to be directly driven by ERK-RSK-S6K. Finally, various transcription factors phosphorylated upon BDNF treatment were previously reported to influence RNA stability, potentially linking the complex RNA regulation revealed by SLAM-seq to the phosphorylation network governed by BDNF.

### Axon-specific RSK and S6K activity is required for BDNF-dependent axonal regeneration

**Figure 6:**
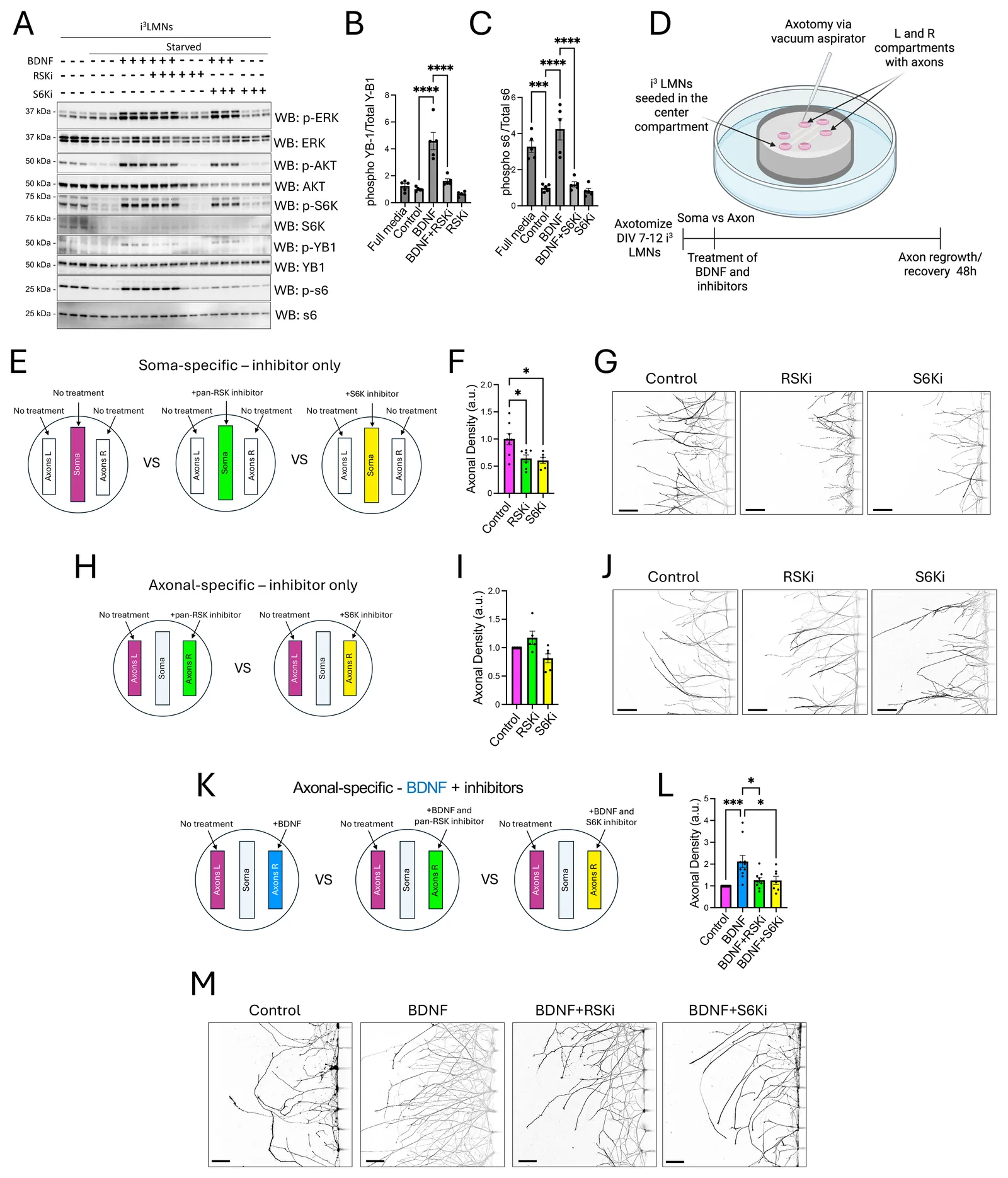
S6K and RSK activation is required for BDNF-dependent axonal regeneration. A) Western analysis of RSK and S6K kinase signalling in i^3^ LMNs after 2h serum starvation and then BDNF treatment (1 h, 25ng/mL), or with co-treatment of S6Ki (PF-4708671, 10uM) or RSKi (LJI308, 1uM). B-C) Quantification of Western analysis in (A). N=5 D) Schematic of microfluidic chambers and aspiration-based axotomy. E) Diagram of experimental comparisons and treatment conditions of axonal regeneration for F-G. F) Quantification of axonal density after axotomy and 48 h recovery with or without RSK inhibitor LJ308 (1 μM) or the S6K inhibitor PF-4708671 (10 μM) in the soma. N = Control 8; RSKi 7; S6Ki 6. G) Representative confocal images of axonal density from F of i^3^ LMNs cultured in tripartite MFCs 48h recovery post-injury. Immunofluorescence of *β*3-tubulin. Scale Bar: 110 µm H) Diagram of experimental comparisons and treatment conditions of axonal regeneration for I-J. I) Quantification of axonal density after axotomy and 48 h recovery with or without RSK inhibitor LJ308 (1 μM) or the S6K inhibitor PF-4708671 (10 μM) in the axon. N = Control 6; RSKi 5; S6Ki 6. J) Representative confocal images of axonal density from I of i^3^ LMNs cultured in tripartite MFCs 48h recovery post-injury. Immunofluorescence of *β*3-tubulin. Scale Bar: 110 µm K) Diagram of experimental comparisons and treatment conditions of axonal regeneration for L-M L) Quantification of axonal density after axotomy and 48 h recovery with or without 50 ng/mL BDNF, RSK inhibitor LJ308 (1 μM) or the S6K inhibitor PF-4708671 (10 μM) treatments in the axons. N = Control 10; BDNF 10; BDNF+RSKi 9; BDNF+S6Ki 7. M) Representative confocal images of axonal density from I of i^3^ LMNs cultured in tripartite MFCs 48h recovery post-injury. Immunofluorescence of *β*3-tubulin. Scale Bar: 110 µm Axonal regeneration experiments were performed using motor neurons derived from at least two rounds of differentiation. Replicates consist of axonal analysis from one microfluidic chamber per condition. For western blotting, each replicate is a well of neurons derived from 2-3 rounds of differentiation. One-way ANOVA, Tukey’s multiple comparison test. Error bars: SEM. p value: ∗ = < 0.05; ∗∗ < 0.01; ∗∗∗ < 0.001; ∗∗∗∗ < 0.00001;

RSKs and S6Ks respond to growth factors through the MAPK and PI3K/AKT pathways (41). Indeed, BDNF robustly activated RSKs and S6Ks kinases as assessed by the phosphorylation of two well-known targets of these pathways, YB-1 and s6, respectively (Fig. 6A-C). Furthermore, YB-1 and s6 phosphorylation were selectively inhibited by a pan-RSK and a S6K inhibitor, respectively. This finding indicates these inhibitors do not cause cross-inhibition. Inhibition of ERK after BDNF treatment led to decreased activation of both RSKs and S6Ks (fig. S4A-C), whereas AKT activity was only required for the S6K signalling arm, but not for RSK, in human motor neurons (fig. S4D-F), indicating that these kinases are activated by BDNF through a canonical pathway.

We next investigated whether RSK and S6K kinase activation was critical for BDNF function in axonal regeneration in human motor neurons. To test this, we cultured i^3^ LMNs in tripartite microfluidic chambers (MFCs) and performed axotomy experiments (Fig. 6D). We axotomized human motor neurons with strong aspiration and allowed the axons to regenerate for 48 hours. First, we tested the effect of inhibiting RSKs or S6K1 in the soma after injury and found that both conditions inhibited axonal regeneration in absence of exogenous BDNF (Fig. 6E-G). Next, we inhibited RSKs and S6K1 only in the axons and found that although both kinases were required in the soma, their basal activation in axons did not affect the basal regenerative capacity of motor neurons (Fig. 6H-J). This indicates that at baseline, the somatic, but not axonal activation of RSK-S6K contributes to axon repair.

Because the two axonal compartments of these MFCs are fluidically isolated from each other and from the somatic compartment, we were able to ascertain the impact of axon-specific treatment of BDNF in one axonal compartment and the simultaneous inhibition of RSK or S6K in the other, in the same motor neuron culture. Axon-specific treatment with BDNF robustly increased axonal regeneration (Fig. 6K-M). This effect of BDNF was blocked when either RSKs or S6K1 were inhibited in axons (Fig. 6K-M). Thus, our data suggest that the upregulation of axonal repair by BDNF depends on the axon-specific activation of RSKs and S6Ks. Furthermore, these experiments functionally link the BDNF-dependent phosphorylation of the substrates reported in Fig. 4-6 to axonal regeneration through RSKs and S6Ks.

### Axonal BDNF enhances axonal regeneration of human motor neurons through localised ERK activation

**Figure 7:**
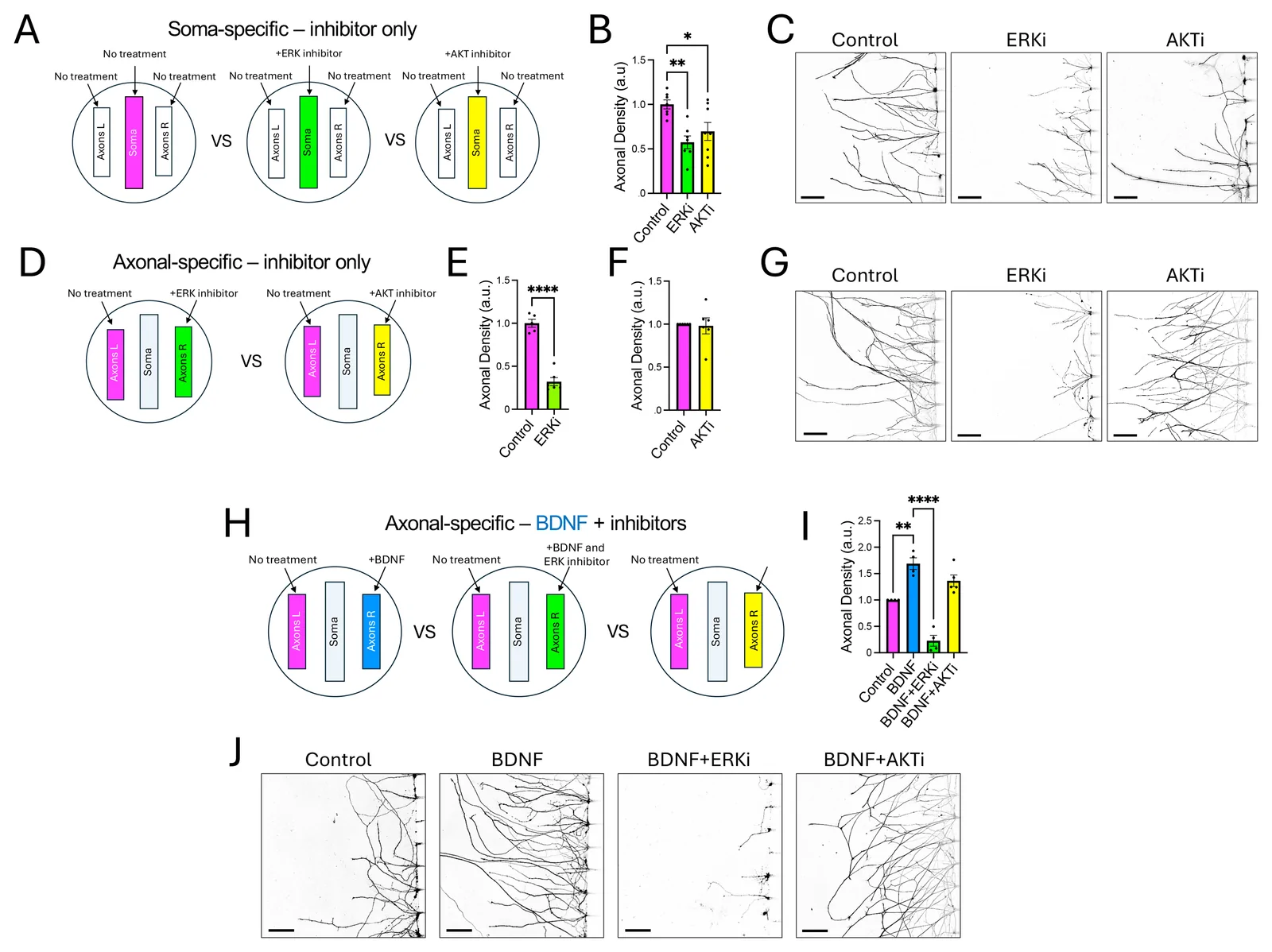
Axon-specific activation of ERK by BDNF enhances regeneration A) Diagram of experimental comparisons and treatment conditions of axonal regeneration for B. B) Quantification of axonal density after axotomy and 48 h recovery with or without treatment with U0126 (20 μM) or ipatasertib (0.1 μM) only in soma. N= Control 7; ERKi 7; AKTi 8 C) Representative confocal images of axons from B. i^3^ LMNs were immunostained with *β*3-tubulin. Scale bars: 110 μm. D) Diagram of experimental comparisons and treatment conditions of axonal regeneration for E-F. E) Axonal density after axotomy and 48 h recovery with or without U0126 (20 μM) treatment of the axonal compartment. N= Control 5; ERKi 6. F) Quantification of axonal density after axotomy and 48 h recovery with or without treatment of ipatasertib (0.1 μM) in the axonal compartment. N= Control 6; AKTi 6. G) Representative confocal images of axons from E-F. i^3^ LMNs were immunostained with *β*3-tubulin. Scale bars: 110 μm. H) Diagram of experimental comparisons and treatment conditions of axonal regeneration for I. I) Quantification of axonal density after axotomy and 48 h recovery with or without BDNF (50 ng/mL) or co-treatment of ERKi U0126 (20 μM) or AKTi ipatasertib (0.1 μM) in the axonal compartment. N= Control 4; BDNF 4; BDNF+ERKi 4; BDNF+AKTi 5. J) Representative confocal images of axons from I. i^3^ LMNs were immunostained with *β*3-tubulin. Scale bars: 110 μm. Axonal regeneration experiments were performed using motor neurons derived from at least two rounds of differentiation. For regeneration assays, replicates consist of axonal analysis from one microfluidic chamber per condition. One-way ANOVA, Turkey’s multiple comparison test. Error bars: SEM. p value: ∗ = < 0.05; ∗∗ < 0.01; ∗∗∗ < 0.001; ∗∗∗∗ < 0.00001.

Lentiviral transduction of TrkB *in vivo* enhances the axonal regeneration of corticospinal neurons though ERK signalling (6). Moreover, RSK and S6K activity are dependent on ERK upon BDNF treatment. Thus, we next explored the requirement for axon-specific activation of ERK by BDNF on axonal regrowth post-injury. To this end, we performed axotomy experiments using i^3^ LMNs cultured in tripartite MFCs under basal conditions. We first tested whether the soma-specific activity of ERK and AKT is required for potentiating regeneration (Fig. 7A). Inhibition of AKT, as well as ERK, in the somatic compartment hindered axonal regeneration post-injury (Fig. 7A-C). To ascertain whether ERK and AKT activity in axons was also required during injury, we treated the axonal compartment with ERK or AKT inhibitors after axotomy. Unlike in the soma experiments, we found that only ERK but not AKT was required for potentiating axonal regeneration through axonal-specific inactivation (Fig. 7D-G). Together these results show that ERK activity is required within axons, while both AKT and ERK are needed in the soma to promote axonal repair. Deletion of PTEN, which negatively regulates the PI3K/AKT pathway, is well-established to foster axonal regeneration (50). Our results showing AKT subcompartment specificity imply that PTEN-inhibition specifically in the soma, but not in the axons, may be driving its pro-regenerative effects.

Next, we asked whether BDNF activation in the axon required the local activity of ERK and AKT. We found the axon-specific effect of BDNF in fostering regeneration was blocked upon ERK inhibition (Fig. 7H-J; fig. S5A-B). These results are in line with our phosphoproteomic analysis showing an enrichment of proline-directed motifs, potentially directed by ERK, on cytoskeletal-binding proteins upon BDNF treatment (Fig. 5; fig. S3A-F). In contrast to ERK inhibition, the axonal-specific inactivation of AKT did not affect BDNF-dependent regeneration (Fig. 7H-J; fig. S5C-D). This striking result was distinct from the data we obtained from RSK and S6K axotomy experiments. Specifically, axonal inactivation of RSK and S6K at baseline do not impair regeneration, yet axonal activation by BDNF of these kinases was necessary for nerve repair of motor neurons. Both ERK and AKT activity were rapidly increased by BDNF, however, ERK displayed a more sustained activation than AKT (fig. S5E-G). Furthermore, we observed quantitative differences in the extent of ERK and AKT activation upon treatment with BDNF in the soma or axonal compartments (fig. S5H-J). Although both kinases are activated in the soma and axons, the activation of ERK was more pronounced in the axon, whereas the AKT was more active in the soma (fig. S5H-J). These results potentially contribute to the compartment-specific effects of inhibiting ERK and AKT in BDNF-dependent axon regeneration.

BDNF-activated TrkB in the periphery is packaged into signalling endosomes and is retrogradely relayed back to the soma where it can then activate kinases locally (51). We therefore asked which kinases activated in the soma by distal stimulation with BDNF were involved in axonal regeneration. To this end, after axotomy, we treated axons with BDNF, and added RSK, S6K, ERK, or AKT inhibitors in the somatic compartment of i^3^ LMNs cultured in MFCs (fig. S7A). We found that, except for RSK, inactivation of all the other kinases lessened the regenerative capacity of axons compared to BDNF alone (fig. S7B-C). This result indicates that although RSK is required in the soma under basal conditions (Fig. 6E-G), axonal BDNF was able to bypass the requirement for somatic RSK to induce axonal regrowth. Furthermore, despite axonal AKT being dispensable for BDNF-dependent axonal regeneration, the activation of AKT in the soma was necessary for axonal BDNF to drive regeneration.

Altogether, our findings reveal the complexity of the spatiotemporal control of kinase activation as it pertains to potentiating axonal repair. We demonstrate that BDNF signalling promotes axonal regeneration by locally activating ERK-S6K-RSK pathway specifically within axons, independent of PI3K/AKT. Thus, BDNF-driven axonal repair relies on a specific subset of kinases, as well as their activation within distinct neuronal compartments.

## Discussion

A central question in the field of axonal regeneration is how neurotrophins enhance nerve repair. Efficient nerve regeneration depends on a complex interplay between intrinsic programs and extrinsic factors. In this study, we reveal that BDNF promotes axonal repair and acts as a signalling hub for multiple neuron-intrinsic regeneration programs in human motor neurons.

Optimal RNA expression relies on the timely coordination of transcription, decay, and stability (52). Transcriptome tuning is particularly crucial for injury response and axonal repair (2, 38). Through SLAM-seq, our findings demonstrate that BDNF exerts highly complex effects on RNA stability in human motor neurons. Unexpectedly, we also found that BDNF drives

hypertranscription, which is not reflected in steady-state RNA levels. This suggests that BDNF calibrates levels of specific RNAs through stabilisation and/or degradation, amidst increased global transcriptional rates. Several RBPs known to regulate RNA stability are top phospho-substrates of BDNF in human motor neurons, which may underlie this phenomenon. Moreover, BDNF regulates the DICER complex and the biogenesis of myriad microRNAs (53). Thus, SLAM-seq revealed a novel regulation of RNA metabolism by BDNF previously unobserved using traditional bulk RNA-seq strategies.

An important question arising from our work is the functional significance of hypertranscription and its relevance to axonal regeneration. Hypertranscription has been shown to facilitate tissue regeneration outside of the nervous system and to accommodate for biosynthetically demanding cellular processes (26). In response to axonal injury, adult neurons revert to embryonic transcriptional states (54) and hypertranscription is more prevalent in developing neurons (26). Thus, performing SLAM-seq after axonal injury has the potential to address whether hypertranscription is a widespread injury-induced response. Additionally, if other neurotrophic factors regulate transcriptional responses through hypertranscription warrants further study. Indeed, there is a paucity of data on the impact of neurotrophic factors on neuronal mRNA stability and decay, which needs to be addressed to fully understand the impact of these neuronal growth factors on neuronal transcriptomes. This is crucial given that stabilisation of RNAs is an emerging paradigm controlling the subcellular localization of RNAs in neurons (8).

The cell-intrinsic capacity of neurons to sustain axonal outgrowth and regeneration require the activation of specific TFs (2, 55). Amidst the profound changes in the transcriptome driven by BDNF, we found that many of the TF-target gene interactions governed by BDNF are enriched for TFs linked to axonal regeneration. We speculate that these TFs function synergistically to orchestrate genes upon BDNF activation (56), fostering nerve repair. Moreover, we find that BDNF rapidly initiates the transcription of injury-inducible RAGs in human motor neurons. Thus, BDNF unlocks the basal cell-intrinsic transcriptional capacity of motor neurons, priming them to respond more aptly to axonal insults. In line with this, genetic manipulations known to improve axonal repair, such as *PTEN* deletion, induce the expression of RAGs independent of axonal injury (35). Future transcriptomic work investigating the effect of BDNF after axonal denervation is warranted to ascertain whether BDNF is also able to transcriptionally attenuate neuronal “death” signals post-injury.

Another aspect that dictates the efficacy of neuron-intrinsic injury response is the modulation of cytoskeletal dynamics (16, 17). We demonstrate that BDNF reshapes the phosphorylation landscape of cytoskeletal-interacting proteins, particularly of structural MAPs and actin-binding proteins. Many of the identified phosphosites are positioned within or adjacent to the cytoskeleton-binding domain, suggesting that BDNF regulates the interaction of these proteins with the cytoskeleton and their function through phosphorylation (18). The global phosphorylation of cytoskeletal-interacting proteins by BDNF may thus underlie the capacity of BDNF to modulate the architecture of multiple neuronal compartments and to foster axonal regeneration. The functional relevance of these novel phosphorylation events to axonal repair and cytoskeletal dynamics requires further exploration, and novel experimental approaches capitalising on reconstituted MAPs and microtubule systems could be an appealing way forward (39).

Our kinase prediction analysis revealed RSKs and S6Ks substrates are enriched upon BDNF treatment. We further show that axon-specific activation of ERK, and its downstream kinases RSKs and S6K are crucial for the axonal regeneration driven by BDNF in human motor neurons. Congruent with our observations, RSKs were previously found to be necessary and sufficient for enhancing axonal regeneration (11, 12). Although our experiments showed that in basal conditions, deactivation of RSK and S6K in the soma but not in axons blocks nerve repair, overexpression of these kinases will be required to fully establish their sufficiency in driving regeneration. Modulating multiple kinases is a strategy at the core of combinatorial therapies to enhance axonal regeneration. Nevertheless, the interrelationship between injury-responsive kinases with respect to boosting regeneration is complex and may yield paradoxical effects (38). For instance, the baseline activity of AKT, RSK and S6K in axons is dispensable for axonal repair. However, axonal activation of RSK and S6K is necessary for BDNF-dependent regeneration. Our data also show that AKT activates RSKs in motor neurons, yet axon-specific activation of AKT by BDNF can be bypassed in terms of its effects in axon repair. Overall, we found that the compartment-specific activation of a subset of kinases by BDNF confers distinct outcomes on axonal repair in human motor neurons.

ERK is activated after peripheral nerve injury to promote axonal regeneration in mice (6, 57) and has well-established roles in cytoskeletal reorganization in axons. RSKs and S6Ks also have a direct impact on microtubule and actin dynamics by phosphorylating specific cytoskeletal components, as inferred from a limited number of studies in mitotic cells (58, 59). Whether the activation ERK-RSK-S6K pathway by BDNF is responsible for the changes in the phosphorylation landscape of cytoskeletal-binding proteins and the modulation of the neuronal cytoskeleton is worth exploring in future studies. It is important to note, however, that the BDNF-dependent phosphosites we identified in cytoskeletal components differ from those found during neuronal maturation (60). This suggests that a transient increase in BDNF affects the phosphorylation status of the cytoskeletal network distinctly from the prolonged process of neuronal maturation.

Neuron-intrinsic pathways boost axonal regeneration through the coordination of transcriptional programs, precise kinase activation patterns, and changes in cytoskeletal dynamics (3, 16, 38). Here, we uncover that BDNF, an extracellular ligand, orchestrates various neuron-intrinsic programs critical for axonal repair. Our data may have substantial implications for the fine-tuning of neurotrophin-based therapies, and novel combinatorial pharmacological interventions targeting both intrinsic and extrinsic regeneration pathways (61), geared towards enhancing axonal viability after traumatic injuries and neuropathies.

## Materials and Methods

### Materials

Materials used in this study, including commercial antibodies, chemicals, reagents, and primers are listed in Data File S5.

### Human iPSC cell culture

A human induced pluripotent stem cell (iPSC) line (WTC11), stably expressing a doxycycline-inducible hNIL construct and CRISPR/Cas9 was used in this study. Briefly, iPSCs were seeded on tissue culture dishes coated with 120-180 μg/ml Geltrex (Thermo Fisher). Cells were maintained in iPSC medium containing Essential 8 Flex Medium (E8F, Thermo Fisher), E8 Flex supplement (Thermo Fisher), and 100 U/ml Pen/Strep and maintained in a 37 ºC, 5% CO2 incubator. Medium change was performed every 1-2 days. Passage of cells were performed with Stem Pro Accutase (Thermo Fisher) for 1-2 min at 37 ºC. Accutase was removed by centrifugation at 300 g for 5 min, and cells were reseeded in iPSC media supplemented with 10 μM ROCK inhibitor (Y-27632, Selleckchem) to facilitate survival. ROCK inhibitor was removed after 24 h.

### i^3^ LMN differentiation from human iPS cells

Differentiation of human iPSCs was performed as previously reported. Briefly, iPSC induction was achieved using induction medium containing DMEM/F12, GlutaMAX supplement medium (Thermo Fisher), MEM non-essential amino acids (Thermo Fisher), N2 supplement (Thermo Fisher), 0.2 mM compound E, 2 μg/ml doxycycline, Pen/Strep and 10 μM Y-27632. After induction for 48 h, cells were dissociated from plates by Accutase and reseeded on poly-D-lysine (PDL)/laminin-coated tissue culture dishes or microfluidic devices in induction medium supplemented with 1 μg/ml laminin. After 24 h, induction medium was replaced by motor neuron medium containing Neurobasal (Thermo Fisher), B27 Plus supplement (Thermo Fisher), N2 supplement, Culture One supplement (Thermo Fisher), 1 μg/ml laminin, 2 μg/ml doxycycline, and Pen/Strep. Medium change was performed every 1-3 days.

### Western blot analyses

Samples for western blot were denatured in lithium dodecyl sulfate (LDS, pH 8.4, NuPAGE) containing 100 mM dithiothreitol (DTT, Sigma) by boiling at 99 ºC for 10 min. The denatured samples were separated on 4-12% Bis-Tris Midi gels (NuPAGE) according to the manufacturer’s instructions. Proteins were transferred to polyvinyl difluoride (PVDF) membranes and immunoblotted using antibodies (1:500 to 1:10,000 in blocking buffer; 1X PBS in 3% milk or bovine serum albumin (BSA)) as specified in the reagent table. For microfluidic chamber western blots, axonal compartments from 3-4 chambers are combined for axonal-enriched lysates.

### Immunofluorescence

Cells were fixed with 4% paraformaldehyde (PFA) in phosphate-buffered saline (PBS) at room temperature for 10 min. Fixed cells were washed with PBS three times. For permeabilization and blocking, cells were incubated with 0.1% saponin, 3% BSA in PBS at room temperature for 1 h. Permeabilized cells were then incubated with 0.05% saponin, 3% BSA in PBS supplemented with antibodies specified in the reagent table overnight at 4 ºC. Cells were then washed with PBS 3 times before incubation with AlexaFluor-conjugated secondary antibodies (Thermo Fisher) at room temperature for 1 h. Cells were then washed with PBS again 5 times before confocal imaging. Images were captured using an inverted Zeiss LSM 980 microscope using a 63x oil-immersed objective.

### Fabrication of tripartite microfluidic devices

The three-compartment microfluidic devices were fabricated using polydimethylsiloxane (PDMS). Moulds with 500 μm long microgrooves were then cured and attached to a PDL-coated Willco glass dishes. After washing with water, the chambers were coated with 50 μg/ml laminin and 0.8% embryonic stem (ES) BSA in DMEM/F12 medium. Prior to seeding, the chambers were washed once with Neurobasal. Differentiated motor neurons (DIV 0 post-differentiation) were seeded into the middle ‘somatic’ compartment with induction media then replaced with motor neuron media after 24 h. Neurons were fed by replacing the motor neuron media every 1-2 days until DIV 12-14 prior to axotomy and imaging experiments.

### Axotomy and axonal regeneration of human motor neuron

Differentiated i^3^ LMNs seeded in three-compartment MFCs were subjected to axotomy at DIV 7-12 post-differentiation when axons reach significant length. Axons were severed by strong aspiration from the axonal compartment and examined under a light microscope to ensure that all axons were severed. MN media or starvation media with BDNF (50 ng/mL), S6Ki (PF-4708671, 10uM), pan-RSKi (LJ308, 1 μM), ERKi (U0126, 20μM), and AKTi (Ipatasertib, 0.1 μM) were then added to the axonal or somatic compartment as specified in figure legends. After 48 h, cells were fixed and processed for immunofluorescence using an anti-b3 tubulin antibody to visualise axons. Confocal microscopy was then performed using a Zeiss LSM 980 (20X objective). For each tripartite microfluidic chamber, at least five images from each of the axonal compartments were acquired. Only fields with axonal regrowth were captured. Using ImageJ, axonal images were binarized and the total area of axons within the region of interest was calculated. Axonal images were then averaged to determine the total area of axonal regrowth in each axonal compartment. For statistical analysis, each microfluidic device was considered as a replicate and neurons from at least 2 differentiation rounds were used to account for batch-to-batch variability of differentiation.

### LC-MS/MS analysis

For analysis on whole cell and phospho-proteomics, cell lysates were digested using the S-trap (ProtiFi) protocol according to the manufacturer’s instructions. Briefly, proteins were reduced using TCEP (10 mM, 30 min, 56 °C) followed by alkylation using iodoacetamide (20 mM, 30 min, under dark). Samples were acidified prior to transfer to the S-trap micro. Proteins were digested in the trap using a trypsin/Lys-C mix. Peptides were cleaned using Sep-Pak C18 minicolumns using manufacturer’s instructions. Cleaned peptide samples were then enriched for phospho-peptides using Titansphere TiO_2_ 5 µm (GL Sciences) beads. Resultant phosphopeptides were then TMT-labelled and a pool of 5% of each TMT-labelled phosphopeptide samples was created and analysed with MS to balance the input amounts for each sample. A pool of equal amounts of each labelled phosphopeptide sample was created and fractionated using a Vanquish HPLC at high pH into 48 fractions. These fractions were dried down and resuspended in 0.1% formic acid (Thermo Fisher) prior to MS analysis. Fractionated phosphopeptides were analysed on a Orbitrap Fusion Lumos Tribrid mass spectrometer interfaced with a 3000 RSLC nano liquid chromatography system. Roughly 1 mg of each sample were loaded onto a 2 cm trapping column (PepMap C18 100A – 300 mm, Thermo Fisher) at 5 µl/min flow rate using a loading pump and analysed on a 50 cm analytical column (EASY-Spray column, 50 cm x 75 μm ID) at 300 nl/min flow rate, that is interfaced to the mass spectrometer using Easy nLC source and electro sprayed directly into the mass spectrometer. A linear gradient between solvent A consisting of 0.1% formic acid in water and solvent B consisting of 0.1% formic acid in 99.9% acetonitrile (Thermo Fisher) was used.

Firstly a gradient of 3% to 30% of solvent B was used at a 300 nl/min flow rate for 105 min and 30% to 40% solvent B for 15 min and 35% to 99% solvent B for 5 min, which was maintained at 90% of B for 10 min and washed the column at 3% solvent B for another 10 min comprising a total of 140 min run with a 120 min gradient in a data dependent acquisition (DDA).

### Mass spectrometry data analysis and bioinformatics analysis

For analysis on whole cell and phospho-proteomics, resultant MS data files were searched using MaxQuant (ver. 2.2.0) against a human database (Uniprot SwissProt database dated February 2022). The search was performed using the default settings with the fixed modification of TMT labels at the MS2 level and the variable modification of Phosphorylation on STY residues. The produced matrix of phosphopeptide abundances was processed using R (ver. 4.1.3) to normalise the data. Missing values were imputed using the Mice R package.

### Kinase activity prediction and scoring

All identified phosphorylation sites were flanked with the seven amino acids upstream and downstream of the phosphosite based on the Uniprot ID. For kinase enrichment, the flanking regions, log_2_fold change and p-value were entered into the `kinase-library` python package (v1.2.0) and prediction was run with `kinase_enrichment` function using the `percentile_rank` method. For site-specific kinase ranking, the flanking regions were used and all sites with a p-value < 0.1 were ranked using the `predict` method. To generate kinase-specific percentile rankings, each kinase’s score for a given peptide was compared against scores from all 311 serine/threonine kinases in the reference panel. This relative ranking positions the kinase within the distribution of all kinases tested against that specific peptide, with the resulting percentile used for visualisation. Python notebooks for running the analyses are available at https://github.com/aleighbrown/lmn_bdnf/.

### RT–qPCR

RNA was extracted from i^3^ LMNs on day 7 after the initiation of differentiation after either 1 hour (*FOS*) or 2 hours (*DYNC1I2*) of BDNF treatment using a RNeasy mini kit (Qiagen) following the manufacturer’s protocol, including the on-column DNA digestion step. RNA concentrations were measured by Nanodrop, and 500 ng of RNA was used for reverse transcription. First-strand cDNA synthesis was performed with RevertAid (Thermo K1622) using random hexamer primers and following the manufacturer’s protocol, including all optional steps. Gene expression analysis was performed by qPCR using PowerUp SYBR Green Master Mix (Thermo A25742) on a QuantStudio 5 or a QuantStudio 6 Flex Real-Time PCR system (Applied Biosystems) and quantified using the ΔΔCt method. *FOS, DYNC1I2*, and *GAPDH* forward and reverse primers are listed in Data File S5. qPCR fold-change data (ΔΔCt values) were log_10_-transformed prior to statistical analysis to achieve approximate normality and satisfy parametric test assumptions. Planned pairwise comparisons between treatment groups were analysed using unpaired t-tests based on specific a priori hypotheses: (1) No treatment vs. BDNF to test stimulatory effects, and (2) BDNF vs. BDNF + actinomycin D/cycloheximide to test transcriptional/translational dependence. Bonferroni correction was applied to account for multiple comparisons.

### SLAM-seq sequencing

SLAM-sequencing was performed on i^3^ LMNs following protocols adapted from Herzog et al. (2017)(20). Three biological replicates of i^3^ LMNs were treated with 100 µM 4sU (Control) + labelling medium or 100 µM 4sU + BDNF (BDNF condition) labelling medium on day 7 for 1 h, 2 h, and 6 h. Cells were washed with PBS and RNA was extracted using the Qiagen RNeasy isolation and purification kit. RNA concentration was measured, and after equilibrating the amount of RNA in each sample, iodoacetamide (10 mM), Na_2_HPO_4_ (pH 8, 50 mM), and DMSO (50% v/v) were added to each sample and incubated at 50 ºC for 15 min to facilitate the thiol-alkylation of 4sU. Samples were processed on RNeasy columns to re-isolate RNA. Sequencing libraries were prepared using the KAPA HyperPrep Kit with RiboErase kit (Roche). Samples were sequenced at 2 x 100 bp on an Illumina NextSeq 2000 machine.

### Total RNA differential gene expression

Sequencing files were trimmed for adapters using Fastp with the parameter “qualified_quality_phred: 10”(62) and quality was assessed using fastqc (63). Quality control examination of sequencing data led to exclusion of one of the four biological replicates being called an outlier for both control and BDNF treated samples, leading to a final N = 3 biological replicates for both controls and BDNF-treated samples. Samples were aligned to the Genome Reference Consortium Human Build 38 (GRCh38) genome using hierarchical indexing for spliced alignment of transcripts - 3 nucleotides (HISAT-3N) (64) and quantified using FeatureCounts using gene models from GENCODE v34. For the differential expression analysis of the total RNA, all samples were run using the standard DESeq2 (65) workflow in R. An adjusted p-value < 0.1 and an absolute log_2_ fold cutoff of 0.75 was used to determine genes with changes in abundance on total RNA level. For differential expression of the previously published RNA-seq(28) dataset, consisting of 2 hours BDNF treated H9 human embryonic stem cell-derived GABAergic inhibitory neurons, we downloaded the count and metadata matrices provided on ArrayExpress (accession no. E-MTAB-6975) and input into the standard differential expression workflow. For the gene-set enrichment analysis on the various axonal injury gene sets for input, to account for variance, we used ‘apeglm’ to shrink log_2_ fold changes prior to the gene-set enrichment analysis.

### New-to-Total RNA labelling analyses

Globally refined analysis of newly transcribed RNA and decay rates using SLAM-seq (GRAND-SLAM) was used to compute the proportion and corresponding posterior distribution of new and old RNA for each gene(24). To test the difference in the fraction of newly transcribed RNA in BDNF and control conditions, we used a Monte-Carlo sampling model. For each gene, we first selected samples where the range of the upper and lower estimate of the newly transcribed ratio was less than 20%. If a gene did not have at least 2 of 3 samples passing this threshold, we did not assess whether the gene was differentially transcribed. Next, we generated a condition probability density for the newly transcribed ratio by generating a beta distribution from all passing samples per condition and inversely weighting each sample by its variance. Then we sampled 1 million times from each condition-specific beta distribution and compared whether the sample was greater in BDNF versus control. Genes were categorised by comparing the fraction of the 1 million samples from each beta distribution where the estimate of new RNA in BDNF condition was higher than the estimate sampled from the control. If 90% of the sampled new RNA estimates was higher in the BDNF compared to Control, then a gene was categorised as ‘bdnf_higher_new_rna’, if less than 10% of samples was higher in BDNF, then a gene was categorised as ‘bdnf_lower_new_rna’, if the BDNF estimate was higher in 40% to 60% of the sampled new RNA estimates, the gene was categorised as ‘bdnf_equals_control’. To control for potential confounding effects of dropout, where highly labelled RNA is underrepresented in metabolic labelling sequencing due to misalignment during analysis (66), we used specialized alignment software (HISAT-3N).

### Total gene expression clustering

Genes were hierarchically clustered by the total RNA log_2_ fold change across time points using ‘ward.D2’ method from the R stats package, and the optimal number of clusters was chosen based on a visual assessment of the cluster plot. Specifically, the plot was examined to identify the number of clusters that demonstrated the highest level of cohesion among members while maintaining a distinct separation from the other clusters.

### Transcription Factor Enrichment Analysis

Gene regulatory networks (GRN) from DoRothEA TF database (37) were first filtered to include only TF-target interactions with confidence levels A. These are the TF-target interactions with the highest level of confidence with multiple lines of evidence supporting the directional interaction, including ChIP-seq, TF binding motif on promoter, manual curation by experts in reviews, or supported in at least two curated resources. We also filtered for GRN with a minimum set size of 5 genes. DoRothEA reports both positive and negative regulatory targets and these GRN were kept as separate gene sets. To control for gene expression significance, we used as input to the GRN all genes ranked according to their p-log10 transformed pvalue signed by the direction of change. GSEA was performed using the ‘fgsea’(67) package in R. Full analysis available in Data File S3. Significant GSEA was called with an adjusted p-value < 0.05.

### Gene-ontology enrichment analyses

All gene-ontology enrichment was performed in R using the clusterProfiler (68) package. In the GO analysis combining total and NTR information, we did not include the GO genes which were either “not significant” on total RNA or where we could not accurately assess differential NTR based on the criteria laid out in the methods section on NTR labelling. To ensure that enriched GO terms would pick up pathways specific to the effects of BDNF treatment and not terms related to tissue-specific expression, only genes which were detected by RNA-seq in the i^3^ LMNs were used as the background gene set. Full GO term analyses are available in Data File S3.

### Gene set enrichment of neuronal regeneration datasets

We identified three gene sets associated with neuronal regeneration. From the Jacobi 2022 paper, we used the 306 genes used to create a ‘composite rag score’ which were selectively expressed in regenerating retinal ganglion found in Data File S4 of the paper (35). From the Chandran 2016 paper, we selected genes from the ‘magenta’ module which were identified as having increased abundance after peripheral nerve injury from Data S2 of the paper (34). From the Renthal 2020 paper, we selected the genes which were common injury genes across neuronal subtypes in Data S4 of the paper (36). We then performed GSEA against these gene sets using both the change in transcription rate, as measured by the log_2_ fold change of the NTR between BDNF and control, and the change in RNA abundance, as measured by the shrunken log_2_ fold change of total RNA.

### Principal component analysis

For total RNA, count data for genes was obtained as described in the method section on *Total RNA differential gene expression*. For labelled RNA, we used the output of GRAND-SLAM which reports the number of reads with at least 2 characteristic conversions as input for count data. Count data was transformed and normalised using in the same manner for total and labelled RNA. First count data was transformed and normalised with the variance-stabilising transformation (VST) implemented in DESeq2 (65). To focus our analysis on the most informative genes, we filtered for the top 500 genes with the highest variance before performing principal component analyses (PCA). PCA and gene loading plots, which illustrate the relative contribution of each gene to a given principal component, were generated from normalised count matrices.

### Statistical analysis

Western blots are analysed using BioRad Image Lab to acquire band intensities. Image J was used to acquire axonal density for regeneration experiments. For both, GraphPad Prism 6 software was used for all statistical analyses. One-way ANOVA was used, unless otherwise specified. All figure legends contain details of statistical tests and sample sizes. At least 2 different batches of differentiation were performed to acquire the i^3^ LMNs used in these experiments to control for batch-to-batch variability of neurons.

## Supplementary Material

Supplementary Materials

## Figures and Tables

**Table 1:** Transcription factors identified by RNA-sequencing associated with axonal regeneration

Transcription Factor	Role in Neuron-Intrinsic Regeneration Pathways	References
**STAT3**	Triggers neuron-intrinsic regeneration pathways in response to axonal injury	(70)
**TP53**	Involved in neuronal regeneration pathways	(71, 72)
**CREB**	Activates regeneration-associated pathways after injury	(73)
**ELK1**	Regulates axonal regeneration after injury	(74)
**SP1**	Facilitates neuronal regeneration processes	(34)
**SRF**	Promotes axonal growth and regeneration	(75)
**ETS1**	Involved in neuronal injury response	(76)
**Jun**	Plays a key role in axonal injury response	(77, 78)
**ATF3**	Synergizes with Jun, STAT3, and SP1 during neuronal damage, crucial for nerve injury response	(36, 79–81)

## Data Availability

RNA sequencing data are available through the ArrayExpress under accession E-MTAB-14537. The mass spectrometry proteomics data have been deposited to the ProteomeXchange Consortium with the PRIDE, partner repository with the dataset identifier PXD046127. The code used in this study are deposited in https://github.com/aleighbrown/lmn_bdnf. No new resources were generated in this study. BioRender was used to generate illustrations within figures. All other data needed to evaluate the conclusions in the paper are present in the paper or the Supplementary Materials.

## One Sentence Summary

BDNF aids nerve repair by fine-tuning the metabolism of RNA and by changing the building blocks of the nerve cell cytoskeleton.
