# Supplementary material for: Brain-derived neurotrophic factor amplifies neuron-intrinsic programs to enhance axonal regeneration in human motor neurons
Source: Sci Signal. Author manuscript; Available in PMC 2026 Oct 19. (PMC7619598; doi:10.1126/scisignal.adx6752)

S4 of the paper (*36*). We then performed GSEA against these gene sets using both the change in transcription rate, as measured by the log_2 fold change of the NTR between BDNF and control, and the change in RNA abundance, as measured by the shrunken log_2 fold change of total RNA.

Principal component analysis

For total RNA, count data for genes was obtained as described in the method section on *Total RNA differential gene expression.* For labelled RNA, we used the output of GRAND-SLAM which reports the number of reads with at least 2 characteristic conversions as input for count data. Count data was transformed and normalised using in the same manner for total and labelled RNA. First count data was transformed and normalised with the variance-stabilising transformation (VST) implemented in DESeq2 (*65*). To focus our analysis on the most informative genes, we filtered for the top 500 genes with the highest variance before performing principal component analyses (PCA). PCA and gene loading plots, which illustrate the relative contribution of each gene to a given principal component, were generated from normalised count matrices.

Statistical analysis

Western blots are analysed using BioRad Image Lab to acquire band intensities. Image J was used to acquire axonal density for regeneration experiments. For both, GraphPad Prism 6 software was used for all statistical analyses. One-way ANOVA was used, unless otherwise specified. All figure legends contain details of statistical tests and sample sizes. At least 2 different batches of differentiation were performed to acquire the i^3 LMNs used in these experiments to control for batch-to-batch variability of neurons.

Supplementary Materials:

fig. S1. i3 LMNs differentiation, axonal development, and response to BDNF treatment.
fig. S2. BDNF differentially regulated genes in human motor neurons.
fig. S3. Phosphorylation patterns of cytoskeletal-binding proteins by BDNF.
fig. S4. BDNF activation of RSK and S6K signalling in human motor neurons.
fig. S5. ERK and AKT signalling in axons of BDNF-treated motor neurons
fig. S6. Axonal-derived BDNF and somatic inhibition of kinases in axonal regeneration
Data file S1. Total and new-to-total differential RNA expression after 1, 2, and 6 hours BDNF treatment.
Data file S2. Gene-cluster assignment, gene-ontology, and transcription factor gene set enrichment.
Data file S3. Differential phosphorylation sites after 1- and 6-hour BDNF treatment
Data file S4. Kinases predicted percentile ranks for identified phospho-sites after 1- and 6-hour BDNF treatment.
Data file S5. Antibody sources and RT-qPCR primers.

Figure legends:

Figure 1. BDNF-driven transcription in human motor neurons regulates time-resolved functional networks

A) Schematic of SLAM-sequencing pipeline. Neurons were treated with 100 µM 4sU uridine analogue which is integrated into newly transcribed RNA. After RNA extraction, RNA was incubated with iodoacetamide, which promotes the thiol-alkylation of 4sU. Before standard RNA-sequencing, RNA is reverse-transcribed to cDNA; during this process the thiol-alkylated 4sU will base pair with guanine rather than adenine, thus mimicking cytosine. When the RNA sequencing is mapped back to the genome, there will be characteristic T > C mismatches in the reads produced during the labelling period which can be used to infer the ratio of new to total RNA. N=3 biological replicates per condition and labelling time.

B) Total RNA principal component analysis showing separation of control (pink) and BDNF treated (blue) lower motor neurons by both treatment condition and time. Close clustering of control samples along PC2 suggests that 4sU treatment had minimal effect on total gene abundance

C) Gene-ontology analyses for genes with increase and decrease in abundance across 1, 2, 6 h BDNF treatment collapsed for semantic similarity. Differentially expressed genes on total RNA called with an adjusted p-value < 0.1 and the abs($\log_2$ fold change) > 0.75 estimated by DESeq2(*65*).

D) Differential gene expression hierarchically clustered by $\log_2$ fold change across BDNF treatment showing mean and individual gene cluster expression with cluster GO terms/gene names. Individual gene expression as red lines, mean expression of cluster dark blue lines, and twice width cluster standard deviation as light blue boundaries. Genes which were never called as differentially expressed at any of the three timepoints and with a baseMean < 15 were removed prior to clustering.

Figure S1. i3 LMNs express motor neuron markers and properly respond to BDNF treatment

A) Schematic of differentiation protocol of i^3 LMNs.

B) Brightfield images of WTC11 iPSC, and DIV1, 3, and 7 i^3 LMNs post-differentiation. Scale bars: 50 µm.

C) Immunofluorescence staining of i3 LMNs for motor neuron markers ChAT and Hb9.

D) Western blot analysis of iPSC and differentiated i^3 LMNs at DIV7 for expression of ChAT.

E) Representation of BDNF signalling pathways (left). Western blot analysis of BDNF signalling in i^3 LMNs after serum starvation for 30 min and BDNF treatment (25 ng/mL) for 10 min.

F) Immunofluorescence of i^3 LMNs (DIV 12) cultured in tripartite MFCs. SMI-31 signal delineates the axonal compartment and MAP2 labels the somatodendritic compartment.

Figure S2. BDNF differentially regulated genes in human motor neurons

A) Gene loading plots for Fig. 1B showing the most important genes for the first 2 principal components. Larger loading values represent a greater contribution to the principal component.

B-D) Volcano plots of differentially expressed genes within 1 h (B) (58 DE genes, 48 increase abundance, 10 decrease abundance), 2 h (C) (2,131 DE genes, 1,172 increase abundance, 959 decrease abundance), and 6 h (D) (604 DE genes, 434 increase abundance, 170 decrease abundance) of BDNF treatment in i3 LMN. Lines define cutoff for a gene being called as differentially expressed (adjusted p-value < 0.1, abs (log2 fold change) > 0.75). Y-axis is cut for genes with p-values approaching zero. N=3 biological replicates per condition and labelling time.

E) GRAND-SLAM estimated NTR and log_{10}-transformed RT-qPCR levels (ΔΔCt method) of *FOS* (1 hour treatment) normalised to GAPDH with BDNF (25 ng/mL) treatment, transcriptional inhibitor actinomycin D (1 µM), and BDNF with actinomycin D. Unpaired t-test with Bonferroni correction for multiple comparisons. Significance: * = < 0.05; ** < 0.01; *** < 0.001; **** < 0.0001. N = 3 biological replicates per condition.

F) Normalised read count and total RNA abundance with log_2fold change and adjusted p-value by DESeq2 and log_{10}-transformed RT-qPCR levels (ΔΔCt method) *DYNC1I2* normalised to GAPDH at 2 hours BDNF (25 ng/mL) treatment, translational inhibitor cycloheximide (100 µg/mL), and BDNF with cycloheximide. Unpaired t-test with Bonferroni correction for multiple comparisons. p value: * = < 0.05; ** < 0.01; *** < 0.001; **** < 0.00001. N = 3 biological replicates per condition.

Figure 2. BDNF coordinates global transcription and widespread changes in RNA stability

A) SLAM-seq converted reads with at least 2 characteristic T>C mismatches per million of mapped reads at 1, 2, and 6 h of 4sU treatment in control and BDNF treated i3 LMN. Schematic of pipeline in Fig. 1A. N=3 biological replicates per condition and labelling time.

B) Converted reads principal component analysis.

C) Schematic of Monte-Carlo sampling approach used to determine condition specific new-to-total RNA ratio (NTR). Beta-distributions on the NTR for a gene for each of N = 3 biological replicates at each time point were estimated by GRAND-SLAM. Condition-specific beta distributions were created by merging the sample beta-distributions with each sample weighted inversely by its variance. Condition NTR was calculated by performing one million samples from each condition's beta distribution, and differential labelling was determined by comparing the difference between each of the 1 million samples.

D) Transcriptome-wide distribution of the log2 fold change of condition specific NTR RNA fraction (left) and the log2 fold change of total RNA. Of genes which met strict NTR cutoff (90% credible interval < 0.2), by 2 h the majority of genes had a higher NTR ratio with BDNF treatment (1 h - 1,392 of 6,169 genes; 2 h - 6,858 of 8,835 genes; 6 h - 8,246 of 8,909 genes).

E) Categorization of genes by BDNF effect on total RNA abundance and NTR across treatment times. Stabilised - increase abundance total RNA, equivalent or lower new RNA; 1 h - 4 genes; 2 h - 123 genes; 6 h - 4 genes. Destabilisation - decrease abundance total

H) Gene-concept heatmap showing the overlap between significantly phosphorylated proteins after 1 h BDNF treatment. The shared gene-ontology terms, which are highlighted in the light blue box, are the GO terms related to cytoskeletal organisation.

Figure S3. Phosphorylation patterns of cytoskeletal-binding proteins by BDNF

A) Sequence logo of differentially phosphorylated sites after 1 h (left) and 6 h (right) of BDNF treatment. The height of letters displays the frequency of the amino acid at each site.
B) Cytoscape StringdB network of BDNF-dependent proline-directed serine residue enrichment for cytoskeletal protein-binding (in yellow). Inputs peptides p-value < 0.05.
C) Western blot analysis of phosphorylated STMN1 (Ser^{25}) in DIV7 i^3 LMNs after 25 ng/ml BDNF with or without the ERK inhibitor U0126 (20 μM) for 1 h.
D) Quantification of the western blot analysis shown in (C); N=7.
E) Western blot analysis of phosphorylated LIN28A (Ser^{200}) after 25 ng/mL BDNF with or without the ERK inhibitor U0126 (20 μM) for 1 h.
F) Quantification of the western blot analysis shown in (E); N=5.
Western blot analyses were performed with neurons derived from at least two independent differentiations. One-way ANOVA, Tukey's multiple comparison. Error bars: SEM. p value: * = < 0.05; ** < 0.01; *** < 0.001; **** < 0.00001.

For western analysis, each replicate is a well of neurons derived from 2-3 rounds of differentiation. One-way ANOVA, Tukey's multiple comparison test. Error bars: SEM. p value: * = < 0.05; ** < 0.01; *** < 0.001; **** < 0.00001;

Figure 5. Kinase prediction of BDNF phosphosubstrates identifies ERK, S6K, and RSK targeting cytoskeletal proteins downstream of BDNF.

A) Predicted kinase enrichment at 1 h and 6 h after BDNF treatment based on sequence of differentially phosphorylated sites. Dotted line shows significant enrichment at p-value < 0.01. B) ERK, S6K, and RSK kinase specificity on microtubule proteins at 1 h BDNF treatment. The heatmap represents the percentile rank of each kinase against the given peptide across the panel of 311 serine/threonine kinases (*49*). The upper ribbon shows the log_2fold change of the phosphorylation after 1 h BDNF treatment. Sites have been hierarchically clustered based on kinase percentile ranks. C) Significant phosphorylation events on proline-directed (pS/TP) and AGC (RXRXXpS/T) motifs downstream of BDNF treatment on microtubule and actin-binding proteins showing proximity to cytoskeletal-binding regions.

Figure 6. S6K and RSK activation is required for BDNF-dependent axonal regeneration.

A) Western analysis of RSK and S6K kinase signalling in i^3 LMNs after 2h serum starvation and then BDNF treatment (1 h, 25ng/mL), or with co-treatment of S6Ki (PF-4708671, 10uM) or RSKi (LJI308, 1uM).
B-C) Quantification of Western analysis in (A). N=5
D) Schematic of microfluidic chambers and aspiration-based axotomy.

E) Diagram of experimental comparisons and treatment conditions of axonal regeneration for F-G.
F) Quantification of axonal density after axotomy and 48 h recovery with or without RSK inhibitor LJ308 (1 μM) or the S6K inhibitor PF-4708671 (10 μM) in the soma. N = Control 8; RSKi 7; S6Ki 6.
G) Representative confocal images of axonal density from F of i^3 LMNs cultured in tripartite MFCs 48h recovery post-injury. Immunofluorescence of β3-tubulin. Scale Bar: 110 μm
H) Diagram of experimental comparisons and treatment conditions of axonal regeneration for I-J.
I) Quantification of axonal density after axotomy and 48 h recovery with or without RSK inhibitor LJ308 (1 μM) or the S6K inhibitor PF-4708671 (10 μM) in the axon. N = Control 6; RSKi 5; S6Ki 6.
J) Representative confocal images of axonal density from I of i^3 LMNs cultured in tripartite MFCs 48h recovery post-injury. Immunofluorescence of β3-tubulin. Scale Bar: 110 μm
K) Diagram of experimental comparisons and treatment conditions of axonal regeneration for L-M
L) Quantification of axonal density after axotomy and 48 h recovery with or without 50 ng/mL BDNF, RSK inhibitor LJ308 (1 μM) or the S6K inhibitor PF-4708671 (10 μM) treatments in the axons. N = Control 10; BDNF 10; BDNF+RSKi 9; BDNF+S6Ki 7.
M) Representative confocal images of axonal density from I of i^3 LMNs cultured in tripartite MFCs 48h recovery post-injury. Immunofluorescence of β3-tubulin. Scale Bar: 110 μm

Axonal regeneration experiments were performed using motor neurons derived from at least two rounds of differentiation. Replicates consist of axonal analysis from one microfluidic chamber per condition. For western blotting, each replicate is a well of neurons derived from 2-3 rounds of differentiation. One-way ANOVA, Tukey's multiple comparison test. Error bars: SEM. p value: * = < 0.05; ** < 0.01; *** < 0.001; **** < 0.00001;

Figure S4. BDNF activation of RSK and S6K signalling in human motor neurons

A) Western blot analysis of RSK and S6K kinase signalling in i^3 LMNs after serum starvation for 2 h before BDNF treatment (1 h; 25 ng/mL), with or without co-treatment of ERKi (U0126; 20 μM).
B) Quantification of the western blot analysis for phospho-s6 shown in (A). N=5.
C) Quantification of the western blot analysis for phospho-YB-1 shown in (A). N=6.
D) Western blot analysis of RSK and S6K kinase signalling in i^3 LMNs after serum starvation for 2 h before BDNF treatment (1 h; 25 ng/mL), with or without co-treatment of AKTi (Ipatasertib; 0.1 μM).
E) Quantification of the western blot analysis for phospho-s6 shown in (D). N=5.
F) Quantification of the western blot analysis for phospho-YB-1 shown in (D). N=4. For western blotting, each replicate is a well of neurons derived from 2-3 rounds of differentiation. One-way ANOVA, Tukey's multiple comparison test. Error bars: SEM. p value: * = < 0.05; ** < 0.01; *** < 0.001; **** < 0.00001.

Figure 7. Axon-specific activation of ERK by BDNF enhances regeneration

A) Diagram of experimental comparisons and treatment conditions of axonal regeneration for B.
B) Quantification of axonal density after axotomy and 48 h recovery with or without treatment with U0126 (20 μM) or ipatasertib (0.1 μM) only in soma. N= Control 7; ERKi 7; AKTi 8
C) Representative confocal images of axons from B. i^3 LMNs were immunostained with β3-tubulin. Scale bars: 110 μm.
D) Diagram of experimental comparisons and treatment conditions of axonal regeneration for E-F.
E) Axonal density after axotomy and 48 h recovery with or without U0126 (20 μM) treatment of the axonal compartment. N= Control 5; ERKi 6.
F) Quantification of axonal density after axotomy and 48 h recovery with or without treatment of ipatasertib (0.1 μM) in the axonal compartment. N= Control 6; AKTi 6.
G) Representative confocal images of axons from E-F. i^3 LMNs were immunostained with β3-tubulin. Scale bars: 110 μm.
H) Diagram of experimental comparisons and treatment conditions of axonal regeneration for I.
I) Quantification of axonal density after axotomy and 48 h recovery with or without BDNF (50 ng/mL) or co-treatment of ERKi U0126 (20 μM) or AKTi ipatasertib (0.1 μM) in the axonal compartment. N= Control 4; BDNF 4; BDNF+ERKi 4; BDNF+AKTi 5.
J) Representative confocal images of axons from I. i^3 LMNs were immunostained with β3-tubulin. Scale bars: 110 μm.

Axonal regeneration experiments were performed using motor neurons derived from at least two rounds of differentiation. For regeneration assays, replicates consist of axonal analysis from one microfluidic chamber per condition. One-way ANOVA, Turkey's multiple comparison test. Error bars: SEM. p value: * = < 0.05; ** < 0.01; *** < 0.001; **** < 0.00001.

Figure S5. ERK and AKT signalling in axons of BDNF-treated motor neurons

A) Western blot analysis of phospho-ERK1/2 in i^3 LMNs after 2h serum starvation and then treatment of BDNF (25 ng/mL) and co-treatment of ERKi (U0126 20 μM) for 1h.
B) Quantification of western blot analysis in (A) N=5
C) Western blot analysis of phospho-S6K in i^3 LMNs after 2h serum starvation adn then treatment of BDNF (25 ng/mL) and co-treatment of AKTi (Ipatasertib 0.1 μM) for 1h.
D) Quantification of western blot analysis in (B). N=4
E) Western blot analysis of a time course of ERK1/2 and AKT signalling in i^3 LMNs after 2h serum starvation and then treatment of BDNF (25 ng/mL) for 5min to 1h.
F) Quantification of ERK1/2 activation across timepoints from (E). Each dot represents an N. N=5 per time point.
G) Quantification of AKT activation across timepoints from (E). Each dot represents an N. N=5 per time point.
H) Western blot analysis of somatic and axonal proteins procured from i^3 LMNs cultured in MFCs after 2h serum starvation and then treatment of BDNF (25 ng/mL) for 1h in all

compartments. SMI-31 and Histone H3 are used as markers for axonal and somatic enrichment, respectively.

I) Quantification of somatic and axonal activation of ERK1/2 by BDNF from (H).

J) Quantification of somatic and axonal activation of AKT by BDNF from (H).

All western blot analyses are performed with neurons derived from at least two differentiation rounds. One-way ANOVA. Error bars: SEM. p value: * = < 0.05; ** < 0.01; *** < 0.001; **** < 0.00001; ns., not significant.

Figure S6. Axonal-derived BDNF and somatic inhibition of kinases in axonal regeneration

A) Diagram of experimental comparisons and treatment conditions in B

B) Quantification of axonal density after axotomy and 48 h recovery with somatic treatment of RSKi (LJ308 1 μM), S6Ki (PF-4708671 10 μM), ERKi (U0126 20 μM), AKTi (Ipatasertib 0.1 μM) with axon-specific treatment of BDNF (50 ng/mL).

C) Representative confocal images of axons from B. i^3 LMNs were immunostained with β3-tubulin. Scale bars: 110 μm.

Axonal regeneration experiments were performed using motor neurons derived from at least two rounds of differentiation. For regeneration assays, replicates consist of axonal analysis from one microfluidic chamber per condition. One-way ANOVA, Turkey's multiple comparison test. Error bars: SEM. p value: * = < 0.05; ** < 0.01; *** < 0.001; **** < 0.00001.

Tables

Table 1. Transcription factors identified by RNA-sequencing associated with axonal regeneration

Transcription Factor	Role in Neuron-Intrinsic Regeneration Pathways	References
**STAT3**	Triggers neuron-intrinsic regeneration pathways in response to axonal injury	(*70*)
**TP53**	Involved in neuronal regeneration pathways	(*71*, *72*)
**CREB**	Activates regeneration-associated pathways after injury	(*73*)
**ELK1**	Regulates axonal regeneration after injury	(*74*)
**SP1**	Facilitates neuronal regeneration processes	(*34*)
**SRF**	Promotes axonal growth and regeneration	(*75*)

Figure S1. i^3 LMNs differentiation, axonal development, and response to BDNF treatment

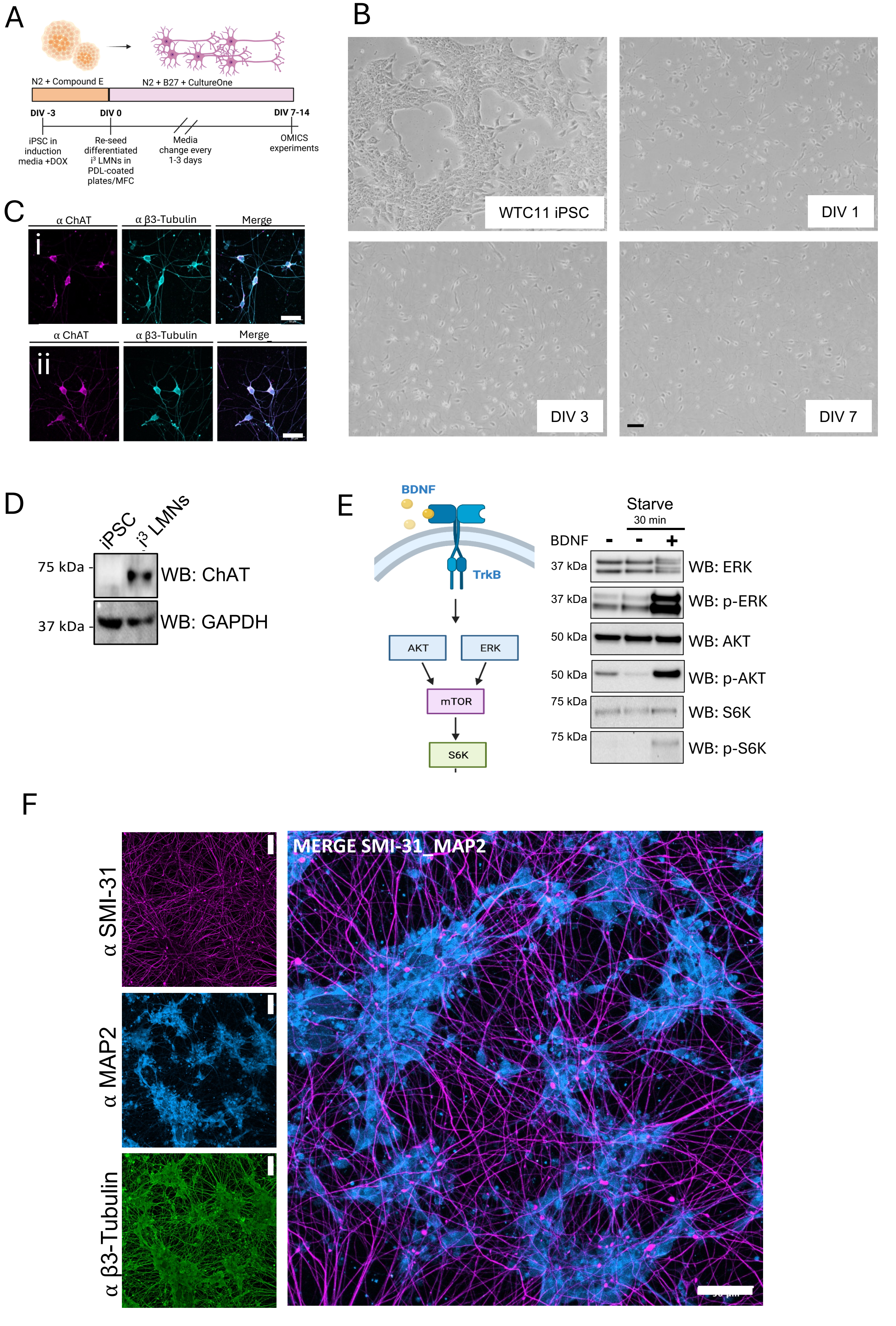

Figure S2. BDNF differentially regulated genes in human motor neurons

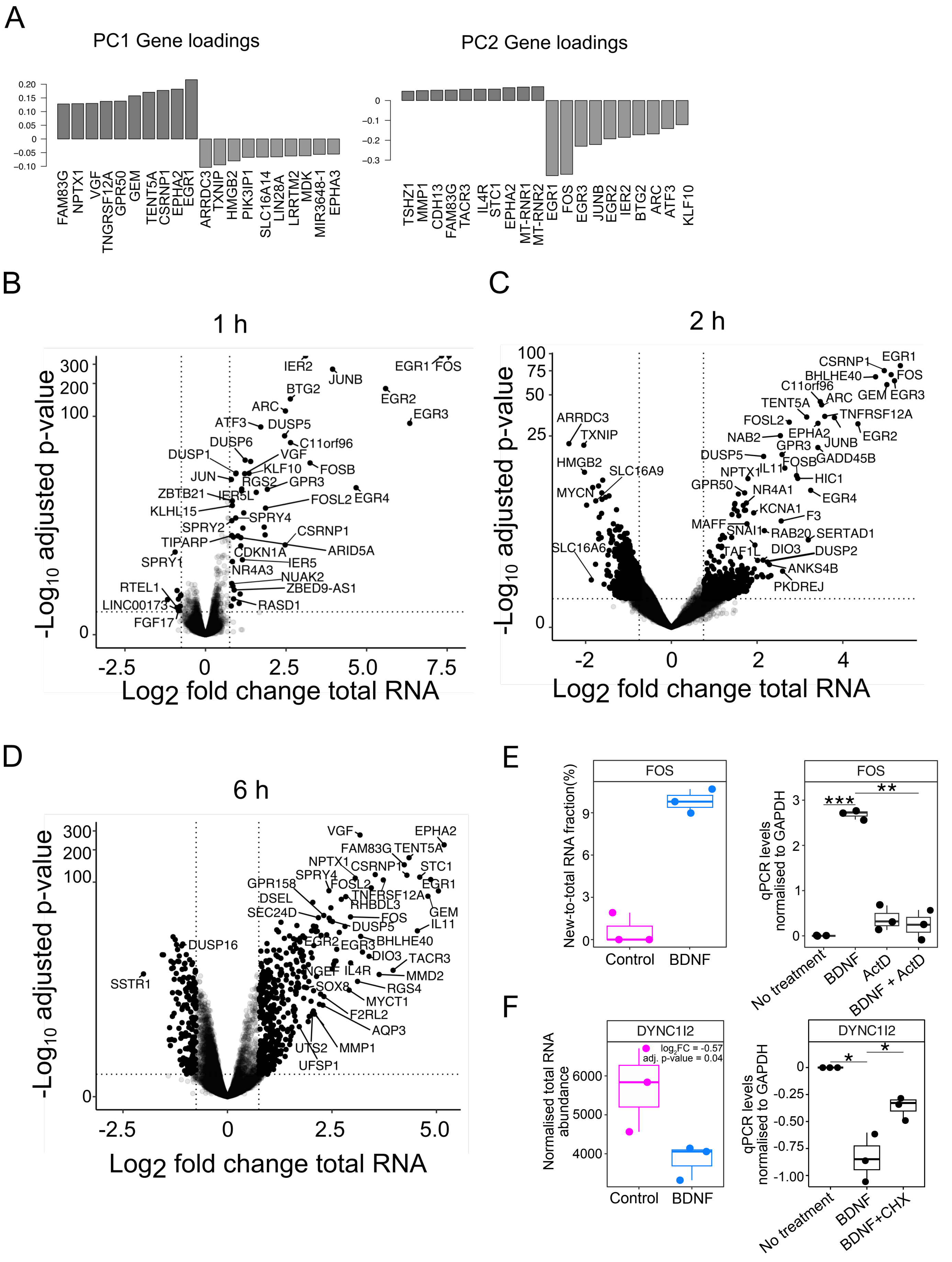

Figure S3. Phosphorylation patterns of cytoskeletal-binding proteins by BDNF

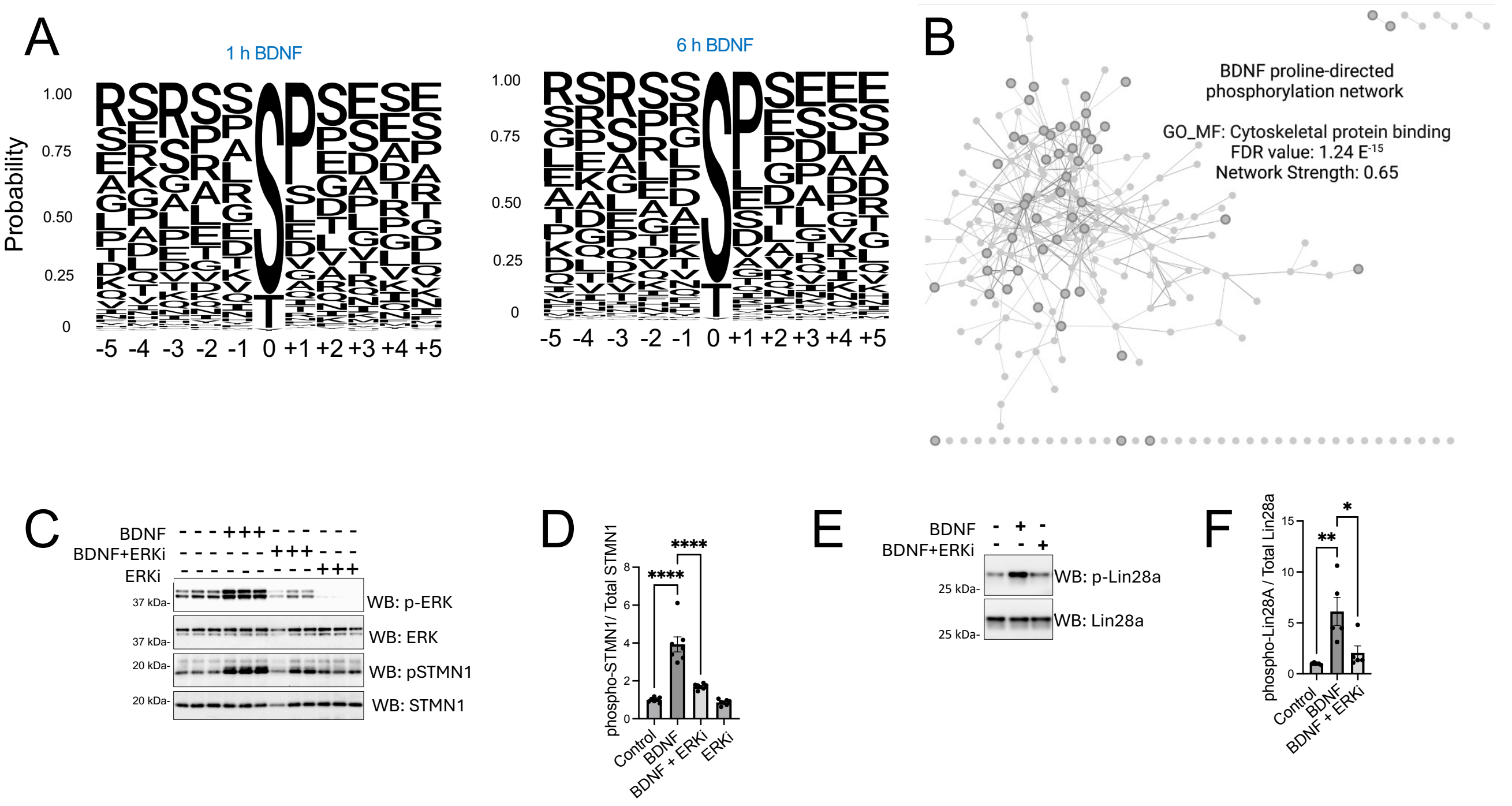

Figure S4. BDNF activation of RSK and S6K signaling in human motor neurons

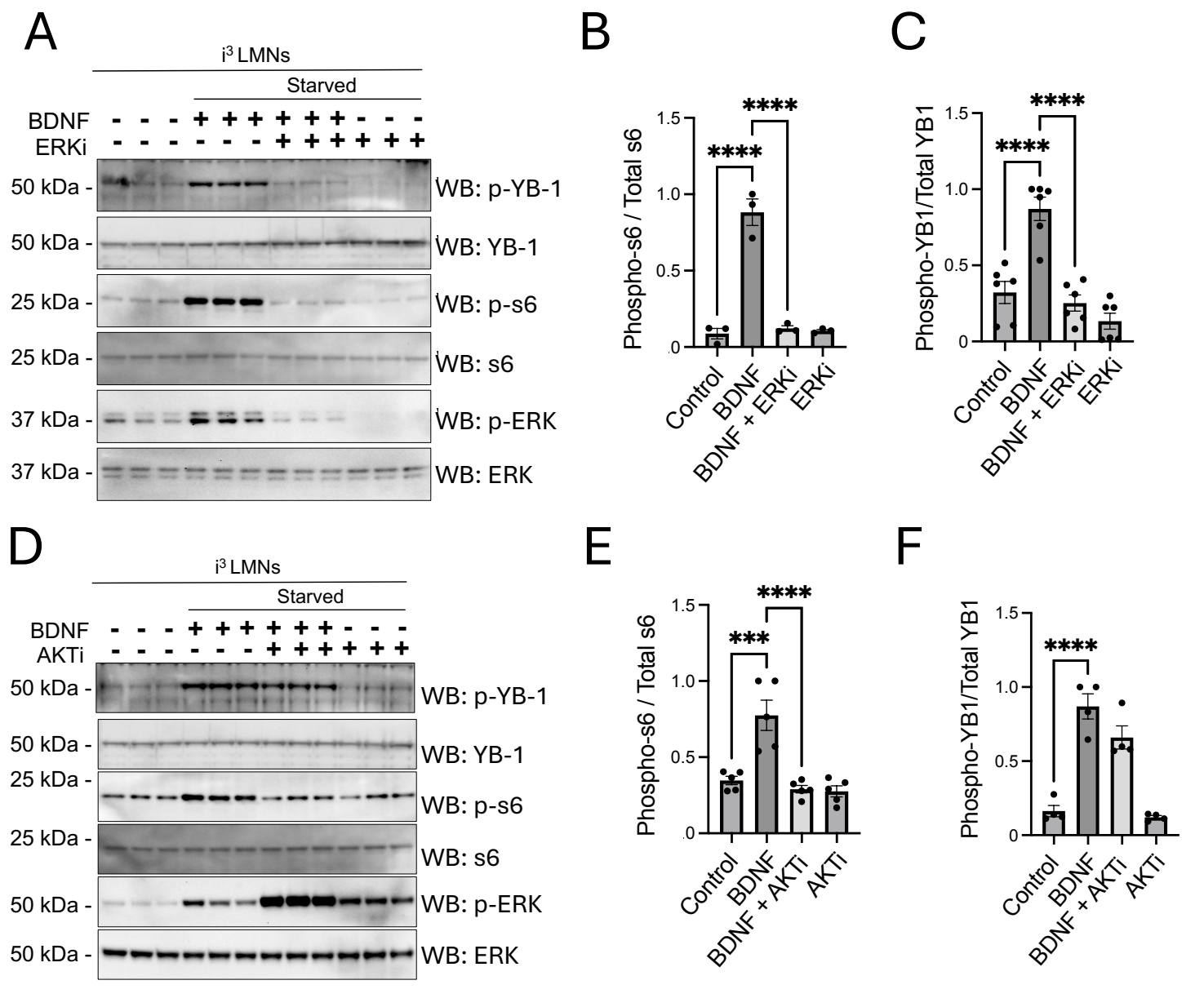

Figure S5. BDNF-driven ERK and AKT signaling in human motor neurons

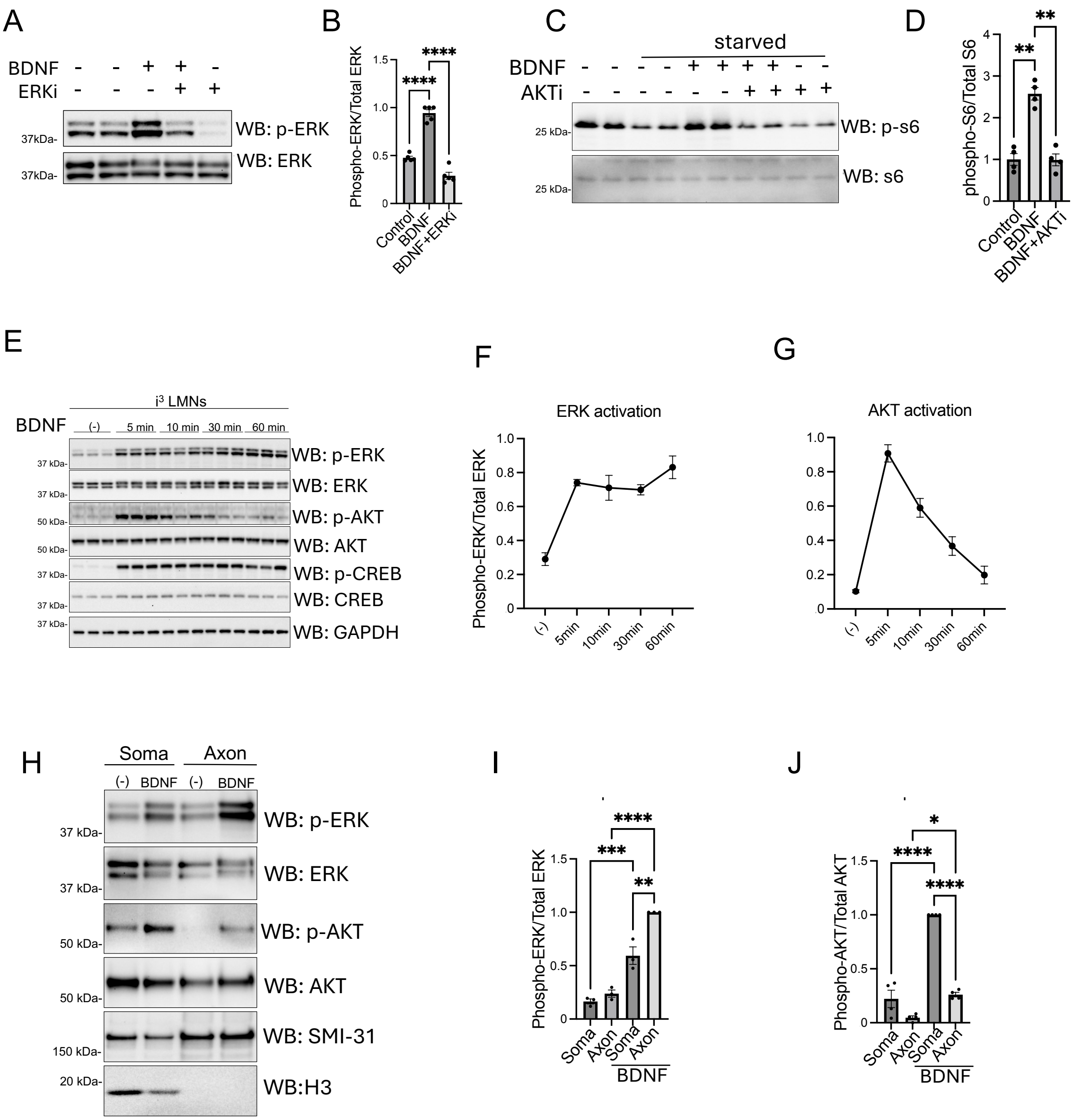

Figure S6. Axonal-derived BDNF and somatic inhibition of kinases in axonal regeneration

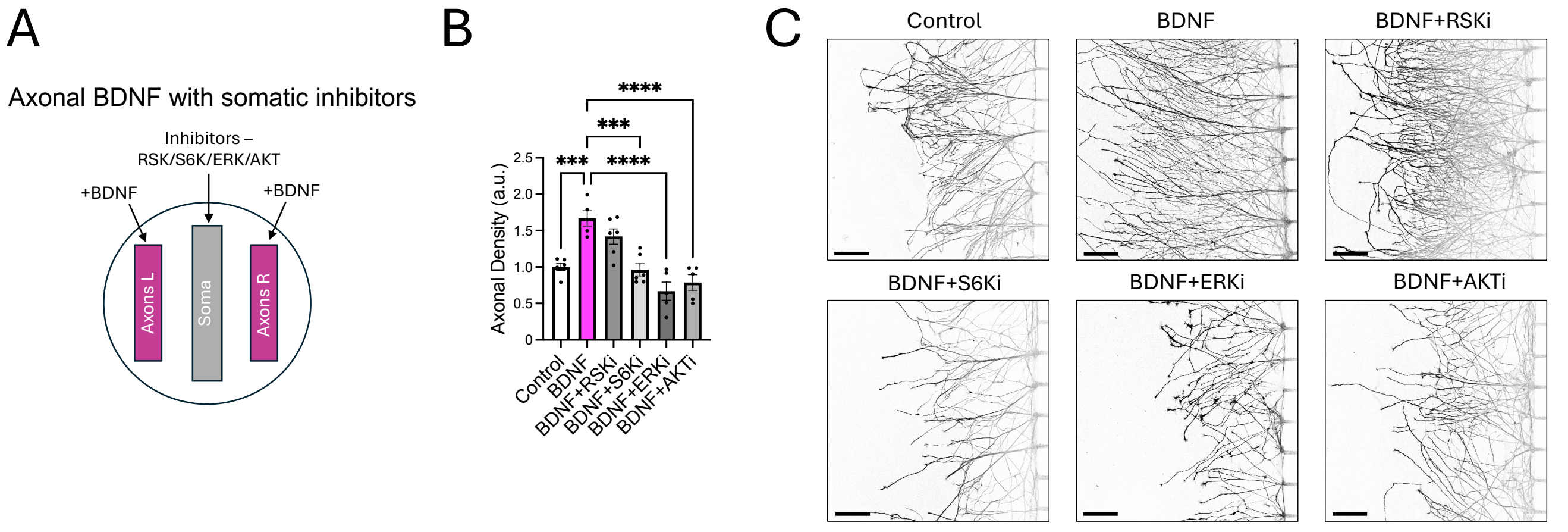

Supplement: Supplementary file 1 [file EMS213867-suppement-Supplementary_Materials.pdf]
